# Development and validation of a blood biomarker score for predicting mortality risk in the general population

**DOI:** 10.1186/s12967-023-04334-w

**Published:** 2023-07-15

**Authors:** Jing Yang, Jiayi Lu, Junyan Miao, Jiacong Li, Meng Zhu, Juncheng Dai, Hongxia Ma, Guangfu Jin, Dong Hang

**Affiliations:** 1 Department of Epidemiology, Center for Global Health, School of Public Health, Nanjing Medical University, 101 Longmian Avenue, Nanjing, 211166 China; 2 Jiangsu Key Lab of Cancer Biomarkers, Prevention and Treatment, Collaborative Innovation Center for Cancer Personalized Medicine and International Joint Research Center on Environment and Human Health, Nanjing Medical University, Nanjing, 211166 China; 3 Research Units of Cohort Study on Cardiovascular Diseases and Cancers, Chinese Academy of Medical Sciences, Beijing, 100730 China

**Keywords:** Biomarkers, Mortality, Risk prediction, Cardiovascular disease, Cancer

## Abstract

**Background:**

Blood biomarkers for multiple pathways, such as inflammatory response, lipid metabolism, and hormonal regulation, have been suggested to influence the risk of mortality. However, few studies have systematically evaluated the combined predictive ability of blood biomarkers for mortality risk.

**Methods:**

We included 267,239 participants from the UK Biobank who had measurements of 28 blood biomarkers and were free of cardiovascular disease (CVD) and cancer at baseline (2006–2010). We developed sex-specific blood biomarker scores for predicting all-cause mortality risk in a training set of 247,503 participants from England and Wales, and validated the results in 19,736 participants from Scotland. Cox and LASSO regression analyses were performed to identify independent predictors for men and women separately. Discrimination and calibration were evaluated by C-index and calibration plots, respectively. We also assessed mediating effects of the biomarkers on the association between traditional risk factors (current smoking, obesity, physical inactivity, hypertension, diabetes) and mortality.

**Results:**

A total of 13 independent predictive biomarkers for men and 17 for women were identified and included in the score development. Compared to the lowest tertile of the score, the highest tertile showed a hazard ratio of 5.36 (95% confidence interval [CI] 5.04–5.71) in men and 4.23 (95% CI 3.87–4.62) in women for all-cause mortality. In the validation set, the score yielded a C-index of 0.73 (95% CI 0.72–0.75) in men and 0.70 (95% CI 0.68–0.73) in women for all-cause mortality; it was also predictive of CVD (C-index of 0.76 in men and 0.79 in women) and cancer (C-index of 0.70 in men and 0.67 in women) mortality. Moreover, the association between traditional risk factors and all-cause mortality was largely mediated by cystatin C, C-reactive protein, 25-hydroxyvitamin D, and hemoglobin A1c.

**Conclusions:**

We established sex-specific blood biomarker scores for predicting all-cause and cause-specific mortality in the general population, which hold the potential to identify high-risk individuals and improve targeted prevention of premature death.

**Supplementary Information:**

The online version contains supplementary material available at 10.1186/s12967-023-04334-w.

## Background

Non-communicable diseases, particularly cardiovascular disease (CVD) and cancer, represent a major threat to public health, accounting for about 73% of all deaths worldwide [1]. Modifiable behaviors, such as tobacco smoking, obesity, and physical inactivity are established risk factors for early death risk [2]. These factors potentially affect multiple pathways, such as inflammatory response, lipid metabolism, liver dysfunction, and hormonal regulation, thereby increasing the risk of morbidity and mortality [3].

Many efforts have been made to identify blood biomarkers associated with mortality, which might be useful to predict the risk of early death. Although the results are not always consistent, several blood biomarkers have been suggested to influence all-cause mortality. For example, C-reactive protein (CRP) [4] and triglycerides (TG) [5] were positively associated, while alanine aminotransferase (ALT) [6] and 25-hydroxyvitamin D (25(OH)D) [7] were inversely associated with all-cause mortality. In addition, sex-specific associations were observed for testosterone in our previous work, showing an inverse association with all-cause mortality in men and a positive association in women [8]. However, the combined performance of these biomarkers to predict death risk in the general population remains largely unknown. Moreover, less is studied for potential mediating effects of these biomarkers on the association between modifiable factors and mortality. An improved understanding of the predictive ability and mediating effects of biomarkers would be useful to identify individuals at high risk of early death and provide tailored prevention strategies.

Therefore, leveraging data from the UK Biobank, a large prospective cohort, we aimed to identify independent predictors of mortality from blood biomarkers involved in multiple pathways, including inflammatory response, lipid metabolism, hormonal regulation, liver and renal function, glucose homeostasis, and bone health. We then developed sex-specific blood biomarker scores and assessed their predictive ability for all-cause and cause-specific mortality. Finally, we performed mediation analysis to quantify the contribution of predictive biomarkers in explaining the associations of traditional risk factors with all-cause mortality.

## Methods

### Study population

UK Biobank is a prospective cohort study recruiting over 500,000 individuals aged 37–73 years from 22 assessment centers across England, Scotland, and Wales between 2006 and 2010 [9]. At recruitment, participants were asked to complete touchscreen questionnaires, have physical measurements taken, and provide biological samples. UK Biobank received ethical approval from North West Multi-Centre Research Ethics Committee (REC reference: 11/NW/03820). All participants signed written informed consent before enrolment.

In the present study, we excluded participants who withdraw from UK Biobank (n = 11), those with prevalent CVD or cancer at baseline (n = 51,323), and those with incomplete data of blood biomarkers (n = 183,931, Additional file 1: Table S1), leaving 267,239 participants in the final analysis. The sample was divided into two sets, a training set including 247,503 participants from England and Wales, and a validation set including 19,736 participants from Scotland (see flowchart in Additional file 7: Fig. S1). The distribution of baseline characteristics between the included and excluded participants did not show significant differences (Additional file 2: Table S2).

### Laboratory tests

Blood samples were collected with standardized procedures and stored at − 80 °C until analysis [10]. A total of 28 biomarkers were assayed, which were implicated in inflammatory response (CRP), lipid and lipid transport (total cholesterol, TG, low-density lipoprotein cholesterol [LDL-C], high-density lipoprotein cholesterol [HDL-C], apolipoprotein A1 [ApoA1], apolipoprotein B), developmental and growth factor (insulin-like growth factor-1 [IGF-1]), sex hormone (testosterone, free testosterone [FT], sex hormone-binding globulin [SHBG]), liver function (ALT, aspartate aminotransferase [AST], gamma-glutamyltransferase [GGT], alkaline phosphatase [ALP], total bilirubin, direct bilirubin, total protein, albumin [ALB]), renal function (cystatin C [CysC], creatinine, urea, urate), glucose homeostasis (hemoglobin A1c [HbA1c], glucose), and bone health (calcium, phosphate, 25(OH)D). All assays were run using internal controls and an external quality assurance scheme. Details about assay methods and quality control procedures are available online (https://biobank.ctsu.ox.ac.uk/crystal/crystal/docs/serum_biochemistry.pdf). The assays were performed on serum samples except that the HbA1c test was performed on packed red blood cells. FT was computed by the validated mass action equation based on SHBG, ALB, and total testosterone concentrations [11, 12]. In addition, serum 25(OH)D concentrations were corrected for seasonal effects by fitting a cosinor model [13]. Because total calcium concentrations vary with the level of ALB [14], ALB-corrected calcium concentrations were calculated [15].

### Assessment of traditional risk factors and other covariates

Information on demographic characteristics, lifestyle factors, and medical history was derived from baseline questionnaires. Traditional risk factors were those previously associated with death risk and confirmed in the UK Biobank, which included tobacco smoking, body mass index (BMI), physical activity, prevalent hypertension, and prevalent diabetes. Current smoking was determined by self-reported smoking status which was categorized into never, previous, or current. BMI was calculated as weight in kilograms divided by height in meters squared. Physical activity was assessed by the total metabolic equivalent of task hours per week. Townsend deprivation index, an indicator of socioeconomic status, was estimated by combining data on housing, employment, and social class based on the postal code of participants [16].

### Ascertainment of outcomes

The date and cause of death were obtained through linkage to national death registries, including the National Health Service (NHS) Digital for participants in England and Wales, and the NHS Central Register for participants in Scotland. Outcomes of interest were classified according to International Classification of Disease edition 10, including mortality due to all-cause, CVD (I00–I79), cancer (C00–D48), respiratory disease (J09–J98), neurological disease (G00–G98), and digestive disease (K20–K93). Detailed information about the linkage procedure and data cleaning is available at https://biobank.ctsu.ox.ac.uk/crystal/refer.cgi?id=115559.

### Statistical analysis

Follow-up time was calculated from the date of recruitment to either the date of death, loss to follow-up, or the end of follow-up (28 February 2021), whichever came first. To improve data normality, biomarker concentrations were natural log-transformed. For the missingness in covariates, we imputed sex-specific median values for continuous variables (all < 22% missing) and used a missing-indicator approach for categorical variables (all < 1% missing). As a sensitivity analysis, we also used the multiple imputation based on chained equations [17] to impute missing covariates for men and women separately. Relevant mediation results remained basically unchanged (data not shown).

We first identified biomarkers that were statistically significantly associated with all-cause mortality in age-adjusted Cox regression models (*P* < 0.05). Then we performed the least absolute shrinkage and selection operator (LASSO) regression analysis with the penalty parameter lambda determined by tenfold cross-validation to select biomarkers of independent predictivity [18]. Sex-specific blood biomarker scores were constructed by a weighted sum of the selected biomarkers, with weights determined by LASSO regression coefficients [19]. To confirm the robustness of the LASSO selection, we also applied random survival forest to select predictors based on variable importance [20] and refitted LASSO regression after excluding participants who died within two years of follow-up or those with abnormal renal function at baseline (creatinine-based estimated glomerular filtration rate < 90 mL/min/1.73 m^2^) [21]. The analyses for biomarker selection and development of the blood biomarker score were conducted exclusively on the training set. We applied the same scoring algorithm in the validation set to assess the accuracy of the score in predicting mortality.

We classified participants into low, intermediate, and high-risk groups according to tertiles of the score and estimated the corresponding 5-, 10-year cumulative probability of death using the Kaplan–Meier method. We also generated a traditional risk score, including tobacco smoking, BMI, physical activity, prevalent hypertension, and prevalent diabetes. For each factor, a low risk level was assigned 1 point and otherwise 0 points. The traditional risk score was constructed as the sum of all five factors, ranging from 0 to 5, with a higher score indicating healthier. Participants were then divided into three groups based on the traditional risk score, i.e., favorable (4–5 points), intermediate (2–3 points), and unfavorable (0–1 points). Calibration was assessed by comparing the deciles of the predicted probability at 10 years with the corresponding observed Kaplan–Meier estimates. Discrimination was assessed by Harrell’s C-index with 95% confidence interval (CI) [22]. We also calculated the C-index when stratifying participants by median follow-up years. In a secondary analysis, we assessed the predictive ability of the blood biomarker score for CVD, cancer, and other causes of death. We also fitted competing risk regression with Fine and Gray subdistribution hazard models as a sensitivity analysis [23].

Furthermore, we conducted mediation analysis in men and women separately to quantify how much of the associations between traditional risk factors and all-cause mortality were mediated through predictive biomarkers by fitting Cox proportional-hazards models both with and without biomarkers [24]. The total effect of traditional risk factors on all-cause mortality were divided into the indirect effect (effect explained by biomarker) and direct effect (effect not explained by biomarker). Each of the traditional risk factors was treated as a dichotomous variable and the lowest risk category was treated as the reference [25].

The risk model development and validation were performed following the TRIPOD guidelines (Additional file 3: Table S3) [26]. All statistical tests were two-sided and performed using SAS (version 9.4) and R (version 4.1.0). *P* < 0.05 was considered as statistical significance.

## Results

During a median follow-up of 12 years, 15,091 deaths occurred, including 7645 from cancer, 2831 from CVD, and 4,615 from other causes. Table 1 shows the baseline demographics characteristics and blood biomarker levels for men and women separately. In the training and validation datasets, men were more likely to be current smokers, physically active, and have a higher prevalence of hypertension and diabetes than women. For blood biomarkers, the median levels of most biomarkers were similar between genders, except that testosterone levels were higher and SHBG levels were lower in men than in women.

**Table 1:** Baseline characteristics of participants stratified by sex in the training and validation sets from the UK Biobank study

	Training set (n = 247,503)		Validation set (n = 19,736)	
	Men (n = 133,834)	Women (n = 113,669)	Men (n = 10,355)	Women (n = 9381)
Age in years, mean ± SD	56.2 ± 8.2	55.3 ± 8.2	55.9 ± 8.1	55.1 ± 8.1
White race, n (%)	126,299 (94)	107,456 (95)	10,171 (98)	9,241 (99)
Townsend deprivation index, mean ± SD	− 1.40 ± 3.04	− 1.44 ± 2.95	− 1.21 ± 3.47	− 1.23 ± 3.39
BMI in kg/m^2^, mean ± SD	27.7 ± 4.2	26.9 ± 5.2	27.6 ± 4.1	26.8 ± 5.1
Physical activity in MET-hours/week, mean ± SD	45.0 ± 45.8	39.3 ± 36.8	42.4 ± 43.4	38.3 ± 35.0
Smoking status, n (%)^a^				
Never	68,076 (51)	69,391 (61)	5606 (54)	5,707 (61)
Previous	49,870 (37)	34,776 (31)	3364 (32)	2785 (30)
Current	15,235 (11)	8991 (8)	1344 (13)	861 (9)
Prevalent hypertension, n (%)	38,188 (29)	26278 (23)	2867 (28)	2087 (22)
Prevalent diabetes, n (%)	7946 (6)	3825 (3)	536 (5)	263 (3)
Blood biomarkers, median (IQR)				
Inflammatory factor				
CRP, mg/L	1.23 (0.64–2.42)	1.23 (0.59–2.63)	1.25 (0.63–2.50)	1.18 (0.56–2.68)
Lipid and lipid transport				
TC, mmol/L	5.48 (4.79–6.18)	5.68 (5.01–6.37)	5.56 (4.87–6.23)	5.72 (5.05–6.40)
TG, mmol/L	1.64 (1.15–2.33)	1.21 (0.89–1.68)	1.63 (1.15–2.35)	1.18 (0.88–1.64)
LDL-C, mmol/L	3.49 (2.95–4.03)	3.47 (2.95–4.02)	3.55 (3.00–4.08)	3.52 (2.99–4.06)
HDL-C, mmol/L	1.25 (1.08–1.46)	1.57 (1.34–1.83)	1.25 (1.08–1.46)	1.57 (1.34–1.83)
ApoA1, g/L	1.41 (1.28–1.57)	1.61 (1.45–1.79)	1.41 (1.28–1.56)	1.61 (1.45–1.79)
ApoB, g/L	1.02 (0.87–1.17)	0.99 (0.85–1.14)	1.03 (0.88–1.19)	1.00 (0.86–1.15)
Developmental and growth factor				
IGF-1, nmol/L	21.9 (18.4–25.3)	21.2 (17.5–24.9)	21.7 (18.3–25.1)	21.0 (17.2–24.7)
Sex hormone				
Testosterone, nmol/L	11.8 (9.6–14.3)	1.03 (0.74–1.40)	12.1 (9.86–14.6)	1.04 (0.75–1.40)
FT, nmol/L	0.15 (0.13–0.19)	0.009 (0.006–0.013)	0.16 (0.13–0.19)	0.009 (0.006–0.013)
SHBG, nmol/L	37.1 (28.1–48.3)	57.6 (41.2–77.9)	37.4 (28.4–48.4)	59.3 (42.8–80.0)
Glucose homeostasis				
HbA1c, mmol/mol	35.0 (32.5–37.6)	34.7 (32.3–37.2)	34.8 (32.4–37.4)	34.4 (32.1–37.0)
Glucose, mmol/L	4.95 (4.61–5.34)	4.90 (4.59–5.25)	4.87 (4.53–5.26)	4.81 (4.50–5.15)
Liver function				
ALT, U/L	23.7 (18.3–31.7)	17.1 (13.6–22.5)	24.8 (19.2–33.2)	17.7 (14.2–23.3)
AST, U/L	26.1 (22.6–30.8)	22.8 (19.8–26.6)	26.2 (22.6–30.8)	22.8 (19.8–26.7)
GGT, U/L	32.4 (23.4–48.8)	20.8 (15.7–30.6)	33.2 (23.5–51.0)	20.8 (15.7–31.2)
ALP, U/L	78.3 (66.7–92.1)	79.6 (65.4–95.8)	79.1 (66.9–93.1)	79.5 (65.5–95.7)
TBIL, μmol/L	9.37 (7.66–11.90)	7.97 (6.72–9.93)	9.37 (7.65–11.84)	7.92 (6.66–9.76)
DBIL, μmol/L	1.79 (1.43–2.28)	1.48 (1.22–1.87)	1.79 (1.43–2.29)	1.48 (1.22–1.86)
TP, g/L	72.5 (69.9–75.2)	72.4 (69.9–75.1)	72.1 (69.7–74.7)	71.8 (69.4–74.4)
ALB, g/L	45.6 (43.9–47.3)	45.1 (43.5–46.8)	45.5 (43.8–47.1)	45.0 (43.3–46.6)
Renal function				
CysC, mg/L	0.91 (0.84–1.00)	0.85 (0.77–0.94)	0.91 (0.83–0.99)	0.85 (0.77–0.94)
Creatinine, μmol/L	80.0 (72.7–88.1)	63.2 (57.3–69.8)	78.9 (71.7–86.5)	62.3 (56.4–68.5)
Urea, mmol/L	5.43 (4.67–6.29)	5.01 (4.27–5.83)	5.41 (4.66–6.26)	4.96 (4.23–5.80)
Urate, μmol/L	348.9 (305.4–396.9)	261.8 (223.6–306.1)	345.3 (300.7–393.3)	257.4 (221.0–302.7)
Bone health				
Calcium, mmol/L^b^	2.36 (2.32–2.41)	2.38 (2.34–2.44)	2.35 (2.30–2.40)	2.37 (2.32–2.42)
Phosphate, mmol/L	1.12 (1.01–1.22)	1.19 (1.09–1.29)	1.08 (0.97–1.19)	1.16 (1.06–1.26)
25(OH)D, nmol/L^b^	47.3 (35.2–61.1)	47.2 (34.7–62.0)	41.1 (31.0–55.1)	41.5 (30.3–56.7)

SD, standard deviation; BMI, body mass index; MET, metabolic equivalent; IQR, interquartile range; CRP, C-reactive protein; TC, total cholesterol; TG, triglycerides; LDL-C, low-density lipoprotein cholesterol; HDL-C, high-density lipoprotein cholesterol; ApoA1, Apolipoprotein A1; ApoB, Apolipoprotein B; IGF-1, insulin-like growth factor-1; FT, free testosterone; SHBG, sex hormone-binding globulin; HbA1c, hemoglobin A1c; ALT, alanine aminotransferase; AST, aspartate aminotransferase; GGT, gamma-glutamyltransferase; ALP, alkaline phosphatase; TBIL, total bilirubin; DBIL, direct bilirubin; TP, total protein; ALB, albumin; CysC, cystatin C; 25(OH)D, 25-hydroxyvitamin D

^a^The totals did not sum to 100% due to small proportions of participants choosing “prefer not to answer”

^b^25(OH)D was adjusted for seasonality and calcium was adjusted for albumin

In the age-adjusted Cox regression models (Additional file 4: Table S4), 23 biomarkers for men and 24 for women were found to be associated with all-cause mortality (*P* < 0.05) in the training set. Based on the LASSO analysis, 13 biomarkers for men and 17 for women were selected as independent predictors for all-cause mortality (Additional file 8: Fig. S2 and Additional file 9: Fig. S3). There were 11 common predictors between genders, including CRP, LDL-C, IGF-1, FT, HbA1c, glucose, GGT, ALP, ALB, CysC, and 25(OH)D. SHBG and creatinine were selected only for men, while TG, ApoA1, testosterone, ALT, AST, and calcium were only for women. The predictors identified by random survival forest were generally consistent with those selected by LASSO (Additional file 10: Fig. S4). When excluding the participants who died within the first two years or those with abnormal renal function, the selected variables were essentially consistent with the main analysis (Additional file 11: Fig. S5 and Additional file 12: Fig. S6). The sex-specific blood biomarker scores ranged from − 11.4 to − 2.1 in men and − 1.2 to 6.6 in women. The score distribution was higher in participants who died than in those alive during the follow-up (Additional file 13: Fig. S7).

**Fig. 1:**
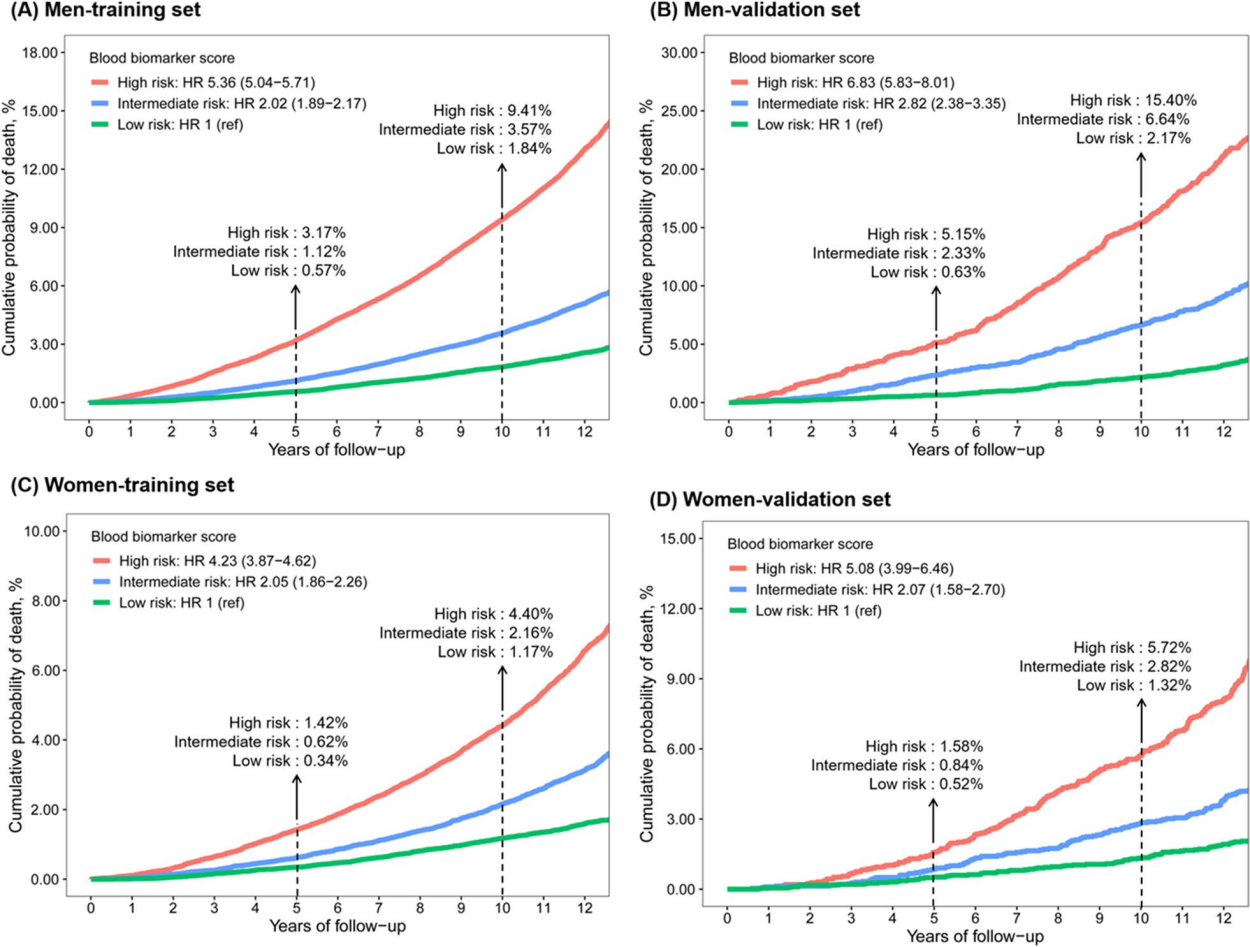
Cumulative probability of death by tertiles of the blood biomarker score in the training and validation sets for men and women

**Fig. 2:**
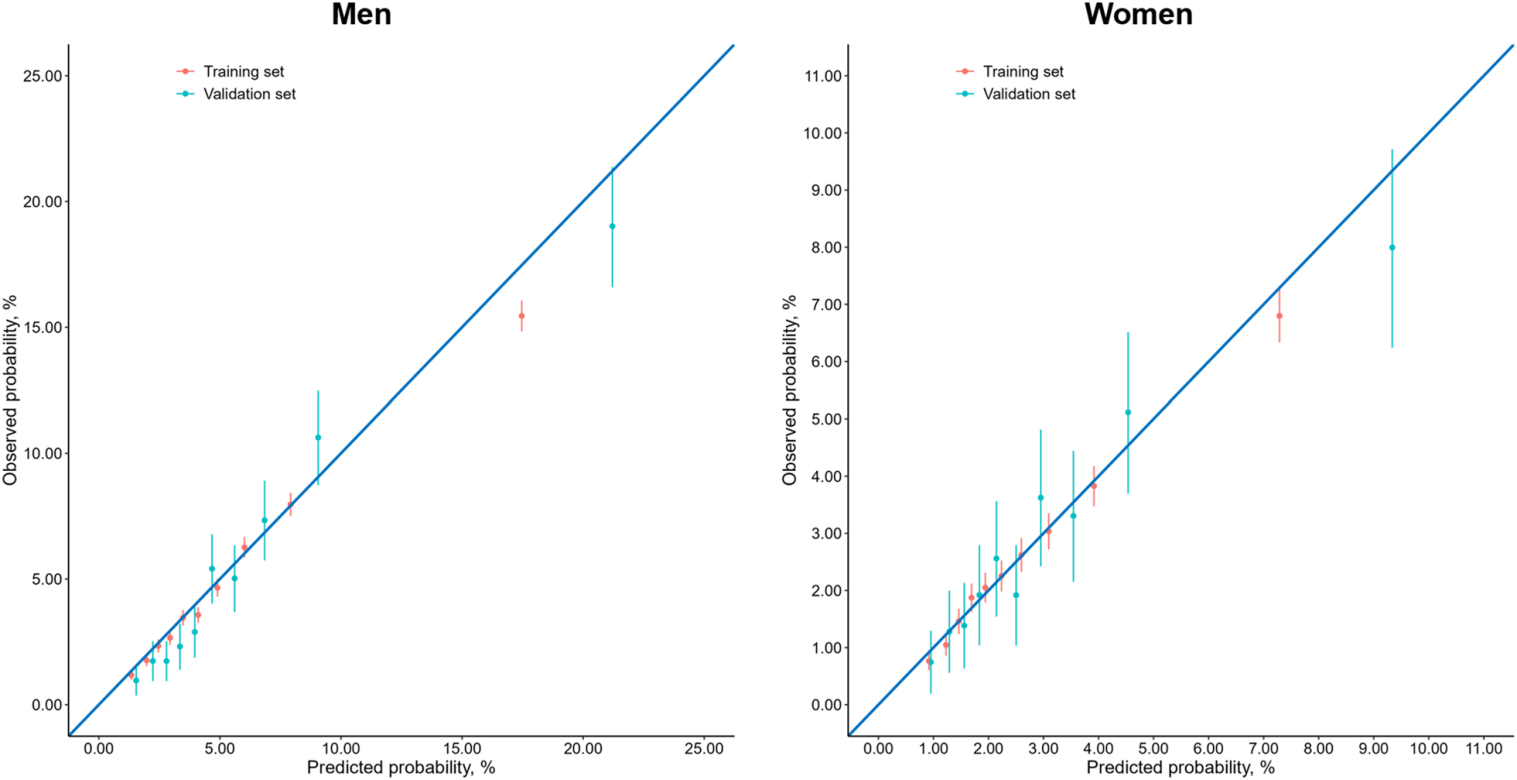
Calibration plots of 10-year all-cause mortality risk

Figure 1 shows the cumulative probability of death according to tertiles of the blood biomarker score. The observed 10-year mortality was 1.84%, 3.57%, 9.41% for men and 1.17%, 2.16%, 4.40% for women correspondingly in low-, intermediate-, and high-risk groups. Using the same cut-offs in the training set, we found that participants in the validation set also showed differentiated all-cause mortality across the risk categories, with the corresponding mortality of 2.17%, 6.64%, 15.40% in men and 1.32%, 2.82%, 5.72% in women, respectively. Compared to the lowest tertile of the score, the highest tertile had a hazard ratio of 5.36 (95% CI 5.04–5.71) in men and 4.23 (95% CI 3.87–4.62) in women for all-cause mortality. The calibration plots for men and women were both well matched with the ideal 45-degree line, indicating good consistency between the predicted and observed estimation of 10-year mortality (Fig. 2). The cumulative probability of death by the three groups of the traditional risk score was plotted in Additional file 14: Fig. S8.

In the training set, the blood biomarker score yielded a C-index of 0.71 (95% CI 0.70–0.71) in men and 0.68 (95% CI 0.67–0.68) in women (Table 2). The traditional risk score had a C-index of 0.59 (95% CI 0.58–0.60) in men and 0.60 (95% CI 0.59–0.61) in women. The predictive performance of the blood biomarker score plus baseline age (C-index of 0.74 in men and 0.72 in women) was also better than that of the traditional risk score plus baseline age (C-index of 0.72 in men and 0.71 in women) (*P* < 0.001). The top 3 biomarkers with high C-index were CysC (0.63), CRP (0.60), and FT (0.60) in men, and CysC (0.65), CRP (0.60), and HbA1c (0.60) in women (Additional file 15: Fig. S9). In the validation set, similar results were observed (C-index of 0.73 [95% CI 0.72–0.75] in men and 0.70 [95% CI 0.68–0.73] in women for the blood biomarker score).

**Table 2:** The C-indices of different risk models for the all-cause mortality prediction

	Training set		Validation set	
	Men	Women	Men	Women
C-index (95% CI)				
Overall				
Blood biomarker score	0.71 (0.70–0.71)	0.68 (0.67–0.68)	0.73 (0.72–0.75)	0.70 (0.68–0.73)
Blood biomarker score plus baseline age	0.74 (0.74–0.75)	0.72 (0.71–0.73)	0.76 (0.75–0.77)	0.76 (0.74–0.78)
Traditional risk score	0.59 (0.58–0.60)	0.60 (0.59–0.61)	0.60 (0.58–0.62)	0.63 (0.60–0.65)
Traditional risk score plus baseline age	0.72 (0.71–0.72)	0.71 (0.70–0.72)	0.73 (0.71–0.74)	0.75 (0.73–0.77)
Combined model^a^	0.75 (0.74–0.75)	0.72 (0.72–0.73)	0.76 (0.75–0.78)	0.77 (0.75–0.79)
*P* value^b^	< 0.001	< 0.001	< 0.001	< 0.001
Follow-up years^c^				
≤ Median				
Blood biomarker score	0.70 (0.69–0.71)	0.67 (0.66–0.68)	0.72 (0.70–0.74)	0.70 (0.67–0.72)
Blood biomarker score plus baseline age	0.73 (0.72–0.73)	0.71 (0.70–0.72)	0.75 (0.73–0.76)	0.75 (0.73–0.77)
Traditional risk score	0.59 (0.58–0.59)	0.60 (0.59–0.61)	0.60 (0.58–0.62)	0.62 (0.60–0.65)
Traditional risk score plus baseline age	0.69 (0.69–0.70)	0.70 (0.69–0.71)	0.71 (0.70–0.73)	0.74 (0.72–0.76)
Combined model^a^	0.73 (0.72–0.73)	0.71 (0.71–0.72)	0.75 (0.73–0.76)	0.75 (0.73–0.77)
> Median				
Blood biomarker score	0.72 (0.69–0.74)	0.66 (0.63–0.69)	0.81 (0.73–0.88)	0.70 (0.59–0.81)
Blood biomarker score plus baseline age	0.75 (0.73–0.77)	0.74 (0.72–0.78)	0.80 (0.71–0.89)	0.73 (0.63–0.84)
Traditional risk score	0.60 (0.57–0.62)	0.62 (0.58–0.65)	0.58 (0.47–0.69)	0.66 (0.53–0.79)
Traditional risk score plus baseline age	0.73 (0.71–0.75)	0.75 (0.72–0.78)	0.70 (0.58–0.83)	0.75 (0.67–0.83)
Combined model^a^	0.75 (0.74–0.77)	0.75 (0.72–0.78)	0.80 (0.71–0.89)	0.75 (0.67–0.84)

*CI* confidence interval

^a^Combined model included baseline age, blood biomarker score, and traditional risk score

^b^The *P* value from a likelihood ratio test comparing the C-indices between blood biomarker score plus baseline age and traditional risk score plus baseline age

^c^For men, the median follow-up years is 11.9 in the training set and 13.1 in the validation set, while for women is 12.0 in the training set and 13.1 in the validation set

**Fig. 3:**
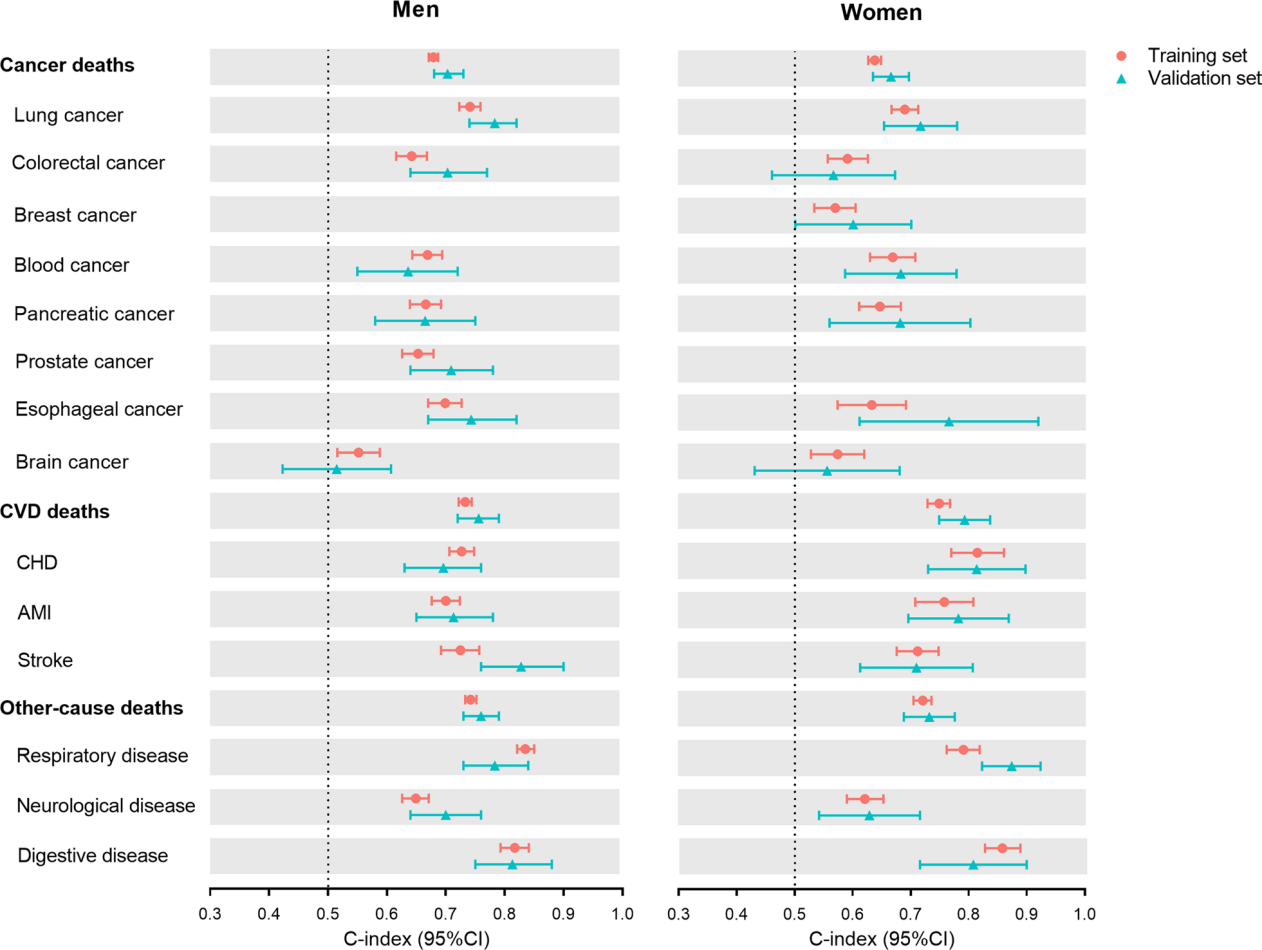
The C-index of blood biomarker scores for cause-specific mortality in the training and validation sets for men and women

We also tested the predictive ability of the score for mortality from CVD, cancer, and other causes (Fig. 3). In the training set, the C-index of the score for predicting CVD mortality was 0.73 (95% CI 0.72–0.74) for total CVD, 0.70 (95% CI 0.68–0.72) for myocardial infarction, 0.73 (95% CI 0.71–0.75) for coronary heart disease, and 0.73 (95% CI 0.69–0.76) for stroke in men. Corresponding values were 0.75 (95% CI 0.73–0.77), 0.76 (95% CI 0.71–0.81), 0.82 (95% CI 0.77–0.86), and 0.71 (95% CI 0.68–0.75) in women, respectively. For cancer mortality, the C-index in men was 0.68 (95% CI 0.67–0.69) for total cancer, ranging from 0.55 (95% CI 0.52–0.59) for brain cancer to 0.74 (95% CI 0.72–0.76) for lung cancer, and in women was 0.64 (95% CI 0.63–0.65) for total cancer, ranging from 0.57 (95% CI 0.53–0.61) for breast cancer to 0.69 (95% CI 0.67–0.71) for lung cancer. In addition, the C-index was 0.74 (95% CI 0.73–0.75) for total other causes, 0.84 (95% CI 0.82–0.85) for respiratory disease, 0.65 (95% CI 0.63–0.67) for neurological disease, and 0.82 (95% CI 0.79–0.84) for digestive disease in men. Corresponding values was 0.72 (95% CI 0.71–0.74), 0.79 (95% CI 0.76–0.82), 0.62 (95% CI 0.59–0.65), and 0.86 (95% CI 0.83–0.89) in women. Similar results were observed in the validation set. The sensitivity analysis using competing risk models showed that the C-indices were only mildly decreased (Additional file 16: Fig. S10).

**Fig. 4:**
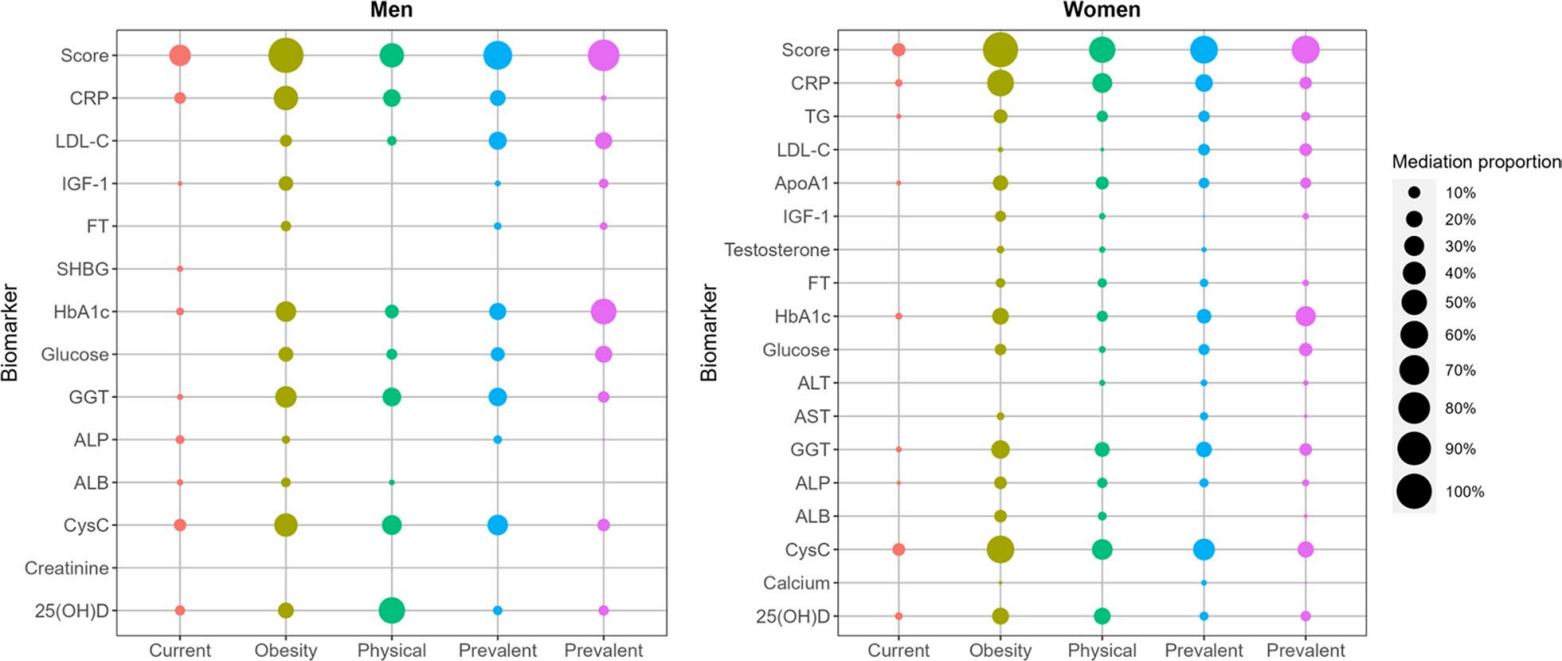
Mediation effects of predictive biomarkers for the associations between traditional risk factors and all-cause mortality among men and women. Mediation analyses were adjusted for age, ethnicity, and Townsend deprivation index. Traditional risk factors were entered as dichotomous variables. The reference group was non-current smoking, non-obesity (BMI < 30 kg/m^2^), physically active (MET-hours/week > median value), non-prevalent hypertension, and non-prevalent diabetes, respectively

In the mediation analysis (Fig. 4, Additional file 5: Table S5, and Additional file 6: Table S6), the blood biomarker score showed significant mediating effects on the associations between traditional risk factors and all-cause mortality, with a maximum proportion of 99.1% in men and 100% in women both for obesity. In men, CysC was the strongest mediator for the associations of current smoking (10.8%) and hypertension (32.1%) with all-cause mortality; CRP, 25(OH)D, and HbA1c mediated the largest effect on obesity (46.2%), physical inactivity (54.1%), and prevalent diabetes (50.9%), respectively. In women, CysC was the strongest mediator for the associations with current smoking (11.4%), obesity (60.1%), physical inactivity (32.6%), and hypertension (36.3%).

## Discussion

In this large-scale prospective study, we identified 13 blood biomarkers in men and 17 biomarkers in women for creating sex-specific biomarker scores that could predict the risk of all-cause and cause-specific deaths. Moreover, we found that the biomarkers, particularly CysC, CRP, 25(OH)D, and HbA1c, exerted significant mediating effects on the associations between traditional risk factors and all-cause mortality. Therefore, our findings provide a panel of biomarkers for improved identification of individuals at high risk of premature death and potential high-priority targets for early prevention.

### Predictive value of blood biomarker score

Up to date, only a small number of studies have assessed the performance of blood biomarkers for predicting death risk. Among 3321 participants from the Cardiovascular Health Study, Sanders et al. derived and validated a biomarker index of five aging-related biomarkers (IGF-1, IGFBP-3, IL-6, DHEAS, and NT-proBNP), showing a C statistic of 0.66 for all-cause mortality [27]. Leveraging data from 3,209 participants in the Framingham Heart Study, Wang et al. constructed a score comprising five biomarkers (CRP, N-terminal pro-atrial natriuretic peptide, homocysteine, renin, and D-dimer) to predict all-cause mortality, with a C statistic of 0.79 for the model including age, sex, and the score [28]. In another study examining the predictivity of 11 blood biomarkers (calcium, BUN, bilirubin, ALB, hematocrit, leukocyte count, uric acid, iron, GGT, ALP, and lactate dehydrogenase) for all-cause mortality in two cohorts, adding the biomarkers into a model of traditional risk factors yielded a validated C statistic of 0.76 [29]. In our study, we excluded participants with prevalent CVD or cancer at baseline to reduce confounding by preexisting diseases. Beyond CRP, calcium, ALB, GGT, ALP, and CysC which have been commonly assessed in previous studies, we additionally included biomarkers from other biological pathways, including lipid metabolism, hormonal regulation, and glucose homeostasis, which probably better reflect the complex and multifactorial nature of death. The results showed that the blood biomarker score had good discriminant power with a C-index of 0.73 for men and 0.70 for women in the validation set. Moreover, our findings indicate that the blood biomarker score has the potential to enhance predictive ability of the traditional risk score for mortality. Of note, although traditional risk factors are easier to be measured, individuals are more likely to modify their unhealthy lifestyles when they become aware of abnormal biochemistry indices. This suggests that the blood biomarker score is a valuable tool in motivating lifestyle changes.

Because of the differences in blood biomarker concentrations across genders, we initially performed sex-specific analyses and revealed that the associations for the majority of biomarkers were consistent between genders, except for total testosterone, FT, SHBG, TG, ALT, creatinine, urea, urate, and phosphate. Our findings confirmed the results from previous studies [30, 31], showing that testosterone was inversely associated, while SHBG was positively associated with all-cause mortality in men. For women, the positive association between testosterone and mortality was also previously reported [32]. In addition, we found that TG, ALT, and urate were positively associated with mortality only in women; creatinine and urea were negatively, while phosphate was positively, associated with mortality only in men. In support of our findings, a meta-analysis reported that women had a stronger positive association between TG and all-cause mortality than men [33]. Another study observed that serum urate was positively associated with all-cause mortality in women [34]. Additionally, a meta-analysis showed that the positive association between serum phosphorus and all-cause mortality existed in men only [35]. The mechanism underlying the sex-specific associations remains unknown and warrants further investigation.

Furthermore, the current study extends the applicability of the score to predict mortality risk from CVD, cancer, and other chronic diseases, since the included biomarkers were derived from multiple key pathways that could contribute to the development of these diseases. To the best of our knowledge, current data are scarce on the prediction of disease-specific mortality in the general population. Only one study investigated a combination of 4 biomarkers (troponin I, N-terminal pro-brain natriuretic peptide, CysC, and CRP) among 1135 elderly Swedish men and reported a C statistic of 0.77 for CVD mortality for the model incorporating the biomarkers and established CVD risk factors [36]. By contrast, our blood biomarker score showed acceptable performances in predicting various cause-specific mortality for middle-aged adults. Thus, previous data and ours support that a panel of specific blood biomarkers is useful to improve risk prediction for deaths from common chronic diseases.

### Mediation effects of blood biomarkers

Interrogating biological mediators of the associations between traditional risk factors and mortality is critical to identifying high-priority targets for developing effective prevention strategies. For the first time, the current study revealed that the created biomarker score was a strong mediator for the associations of smoking, obesity, physical inactivity, hypertension, and diabetes with all-cause mortality. Specifically, CysC, CRP, 25(OH)D, and HbA1c accounted for the highest mediation proportions, suggesting that the biomarkers or pathways represented by these biomarkers could explain most of the association between traditional risk factors and early death risk. CysC is commonly used to measure glomerular filtration rate as an index of renal function [37]. In addition, emerging biological evidence suggests that CysC plays a regulatory role in immune response, apoptosis, autophagy, and tumor metastasis [38]. In support of our findings, previous studies revealed CRP as an important mediator for obesity [39], 25(OH)D as a mediator for physical inactivity [40], and HbA1c for diabetes [41], in relation to the risk of common chronic disease. These data suggest that CysC, CRP, 25(OH)D, and HbA1c might represent potential targets for mitigating premature death risk.

### Strengths and limitations

Our study has several strengths, including a large sample size with a training and validation design, a long-term follow-up, inclusion of various biomarkers from multiple pathways, strict control for potential confounding, and detailed sensitivity analysis. However, several limitations need to be noted as well. First, a single measurement of blood biomarkers at baseline may not reflect long-term exposures. However, previous evidence suggests that one-time measurement of the included biomarkers (e.g., CysC, testosterone, and 25[OH]D) could reliably categorize average levels over at least a 4-year period [8, 42, 43]. Second, we were unable to evaluate the potential effect of estrogens on mortality because the assay used in the UK Biobank to assess estradiol levels was not sufficiently sensitive to measure the typically low concentrations in postmenopausal women and men. Third, due to the relatively small numbers of cause-specific deaths in the validation set, the predictive estimates by C-indices with wide CIs should be interpreted with caution. Finally, most of the UK Biobank participants were of European ancestry. The predictive power remains to be validated in other ethnicities from external datasets.

## Conclusions

In conclusion, we developed sex-specific biomarker scores that have the potential to increase prediction accuracy for all-cause and cause-specific mortality. In addition, the biomarkers had significant mediating effects on the associations between traditional risk factors and mortality, which might be potential high-priority targets for early prevention. Further research is required to validate these findings and uncover underlying mechanisms for translating the evidence into practice.

## Supplementary Information

**Additional file 1: Table S1.** The assay methods and missingness of blood biomarkers (n = 451,170).

**Additional file 2: Table S2.** Comparison of baseline characteristics between the included and excluded UK Biobank participants.

**Additional file 3: Table S3.** TRIPOD Checklist—Prediction Model Development and Validation.

**Additional file 4: Table S4**. Associations of blood biomarkers with all-cause mortality for men and women in the training set.

**Additional file 5: Table S5.** Mediation effects of predictive biomarkers for the associations between traditional risk factors and all-cause mortality in men.

**Additional file 6: Table S6.** Mediation effects of predictive biomarkers for the associations between traditional risk factors and all-cause mortality in women.

**Additional file 7: Figure S1.** Flowchart of study population selection.

**Additional file 8: Figure S2.** Biomarker selection using the Least absolute shrinkage and selection operator (LASSO) regression in the training set for men and women. Ten-fold cross-validation for tuning parameter selection in the LASSO regression (A and C); LASSO coefficient profiles of the 28 candidate biomarkers (B and D).

**Additional file 9: Figure S3.** The coefficients of selected biomarkers derived from the LASSO regression for all-cause mortality in the training set.

**Additional file 10: Figure S4.** Variable importance based on random survival forest models for all-cause mortality prediction. The asterisk denotes the variables selected from the LASSO regression in the training set.

**Additional file 11: Figure S5.** The coefficients of selected biomarkers derived from the LASSO regression for all-cause mortality in the training set after excluding 584 men and 259 women within two years of follow-up time.

**Additional file 12: Figure S6.** The coefficients of selected biomarkers derived from the LASSO regression for all-cause mortality in the training set after excluding 54,428 men and 42,998 women with estimated glomerular filtration rate < 90 mL/min/1.73 m^2^.

**Additional file 13: Figure S7.** The distribution of the blood biomarker score between participants dead and alive in the training and validation sets.

**Additional file 14: Figure S8.** Cumulative probability of death by the three groups of traditional risk score in the training and validation sets for men and women.

**Additional file 15: Figure S9.** The C-index of predictors for all-cause mortality in the training and validation sets for men and women.

**Additional file 16: Figure S10.** The C-index of blood biomarker scores for cause-specific mortality using competing risk models in the training and validation sets for men and women.

## Data Availability

UK Biobank is an open access resource, and the study website http://www.ukbiobank.ac.uk/register-apply has information on available data and access procedures.

## Abbreviations

25(OH)D: 25-Hydroxyvitamin D
ALB: Albumin
ALP: Alkaline phosphatase
ALT: Alanine aminotransferase
AMI: Acute myocardial infarction
ApoA1: Apolipoprotein A1
ApoB: Apolipoprotein B
AST: Aspartate aminotransferase
BMI: Body mass index
CHD: Coronary heart disease
CI: Confidence interval
CRP: C-reactive protein
CVD: Cardiovascular disease
CysC: Cystatin C
DBIL: Direct bilirubin
FT: Free testosterone
GGT: Gamma-glutamyltransferase
HbA1c: Hemoglobin A1c
HDL-C: High-density lipoprotein cholesterol
IGF-1: Insulin-like growth factor-1
LDL-C: Low-density lipoprotein cholesterol
LASSO: Least absolute shrinkage and selection operator
NHS: National Health Service
SHBG: Sex hormone-binding globulin
TBIL: Total bilirubin
TC: Total cholesterol
TG: Triglyceride
TP: Total protein
TRIPOD: Transparent Reporting of a Multivariable Prediction Model for Individual Prognosis or Diagnosis

## References

[1] Roth GA, Abate D, Abate KH, Abay SM, Abbafati C, Abbasi N, Abbastabar H, Abd-Allah F, Abdela J, Abdelalim A (2018). Global, regional, and national age-sex-specific mortality for 282 causes of death in 195 countries and territories, 1980–2017: a systematic analysis for the Global Burden of Disease Study 2017. Lancet.

[2] Murray CJL, Aravkin AY, Zheng P, Abbafati C, Abbas KM, Abbasi-Kangevari M, Abd-Allah F, Abdelalim A, Abdollahi M, Abdollahpour I (2020). Global burden of 87 risk factors in 204 countries and territories, 1990–2019: a systematic analysis for the Global Burden of Disease Study 2019. Lancet.

[3] Friedenreich CM, Ryder-Burbidge C, McNeil J (2021). Physical activity, obesity and sedentary behavior in cancer etiology: epidemiologic evidence and biologic mechanisms. Mol Oncol.

[4] Sung KC, Ryu S, Chang Y, Byrne CD, Kim SH (2014). C-reactive protein and risk of cardiovascular and all-cause mortality in 268 803 East Asians. Eur Heart J.

[5] Langsted A, Freiberg JJ, Tybjaerg-Hansen A, Schnohr P, Jensen GB, Nordestgaard BG (2011). Nonfasting cholesterol and triglycerides and association with risk of myocardial infarction and total mortality: the Copenhagen City Heart Study with 31 years of follow-up. J Intern Med.

[6] Ford I, Mooijaart SP, Lloyd S, Murray HM, Westendorp RG, de Craen AJ, Packard CJ, Buckley B, Barlow C, Preiss D (2011). The inverse relationship between alanine aminotransferase in the normal range and adverse cardiovascular and non-cardiovascular outcomes. Int J Epidemiol.

[7] Fan X, Wang J, Song M, Giovannucci EL, Ma H, Jin G, Hu Z, Shen H, Hang D (2020). Vitamin D status and risk of all-cause and cause-specific mortality in a large cohort: results from the UK biobank. J Clin Endocrinol Metab.

[8] Wang J, Fan X, Yang M, Song M, Wang K, Giovannucci E, Ma H, Jin G, Hu Z, Shen H, Hang D (2021). Sex-specific associations of circulating testosterone levels with all-cause and cause-specific mortality. Eur J Endocrinol.

[9] Collins R (2012). What makes UK Biobank special?. Lancet.

[10] Elliott P, Peakman TC, Biobank UK (2008). The UK Biobank sample handling and storage protocol for the collection, processing and archiving of human blood and urine. Int J Epidemiol.

[11] Rinaldi S, Geay A, Déchaud H, Biessy C, Zeleniuch-Jacquotte A, Akhmedkhanov A, Shore RE, Riboli E, Toniolo P, Kaaks R (2002). Validity of free testosterone and free estradiol determinations in serum samples from postmenopausal women by theoretical calculations. Cancer Epidemiol Biomarkers Prev.

[12] Södergård R, Bäckström T, Shanbhag V, Carstensen H (1982). Calculation of free and bound fractions of testosterone and estradiol-17 beta to human plasma proteins at body temperature. J Steroid Biochem.

[13] Sachs MC, Shoben A, Levin GP, Robinson-Cohen C, Hoofnagle AN, Swords-Jenny N, Ix JH, Budoff M, Lutsey PL, Siscovick DS (2013). Estimating mean annual 25-hydroxyvitamin D concentrations from single measurements: the Multi-Ethnic Study of Atherosclerosis. Am J Clin Nutr.

[14] Minisola S, Pepe J, Piemonte S, Cipriani C (2015). The diagnosis and management of hypercalcaemia. BMJ.

[15] Schini M, Hannan FM, Walsh JS, Eastell R (2021). Reference interval for albumin-adjusted calcium based on a large UK population. Clin Endocrinol (Oxf).

[16] Townsend P, Phillimore P, Beattie AJRcdhye: Health and deprivation. Inequality and the North. 1997, 35.

[17] White IR, Royston P, Wood AM (2011). Multiple imputation using chained equations: Issues and guidance for practice. Stat Med.

[18] Tibshirani R (1997). The lasso method for variable selection in the Cox model. Stat Med.

[19] Li J, Guasch-Ferre M, Chung W, Ruiz-Canela M, Toledo E, Corella D, Bhupathiraju SN, Tobias DK, Tabung FK, Hu J (2020). The Mediterranean diet, plasma metabolome, and cardiovascular disease risk. Eur Heart J.

[20] Aleksandrova K, Reichmann R, Kaaks R, Jenab M, Bueno-de-Mesquita HB, Dahm CC, Eriksen AK, Tjonneland A, Artaud F, Boutron-Ruault MC (2021). Development and validation of a lifestyle-based model for colorectal cancer risk prediction: the LiFeCRC score. BMC Med.

[21] Levey AS, Stevens LA, Schmid CH, Zhang YL, Castro AF, Feldman HI, Kusek JW, Eggers P, Van Lente F, Greene T, Coresh J (2009). A new equation to estimate glomerular filtration rate. Ann Intern Med.

[22] Harrell FE, Lee KL, Mark DB (1996). Multivariable prognostic models: issues in developing models, evaluating assumptions and adequacy, and measuring and reducing errors. Stat Med.

[23] Fine JP, Gray RJ (1999). A proportional hazards model for the subdistribution of a competing risk. J Am Stat Assoc.

[24] VanderWeele TJ (2011). Causal mediation analysis with survival data. Epidemiology.

[25] Hertzmark E, Pazaris M, Spiegelman D The SAS mediate macro. 2018.

[26] Moons KG, Altman DG, Reitsma JB, Ioannidis JP, Macaskill P, Steyerberg EW, Vickers AJ, Ransohoff DF, Collins GS (2015). Transparent reporting of a multivariable prediction model for Individual Prognosis or Diagnosis (TRIPOD): explanation and elaboration. Ann Intern Med.

[27] Sanders JL, Arnold AM, Boudreau RM, Hirsch CH, Kizer JR, Kaplan RC, Cappola AR, Cushman M, Jacob ME, Kritchevsky SB, Newman AB (2019). Association of biomarker and physiologic indices with mortality in older adults: cardiovascular health study. J Gerontol A Biol Sci Med Sci.

[28] Wang TJ, Gona P, Larson MG, Tofler GH, Levy D, Newton-Cheh C, Jacques PF, Rifai N, Selhub J, Robins SJ (2006). Multiple biomarkers for the prediction of first major cardiovascular events and death. N Engl J Med.

[29] Lind L, Zanetti D, Hogman M, Sundman L, Ingelsson E (2020). Commonly used clinical chemistry tests as mortality predictors: Results from two large cohort studies. PLoS ONE.

[30] Tivesten A, Vandenput L, Labrie F, Karlsson MK, Ljunggren O, Mellström D, Ohlsson C (2009). Low serum testosterone and estradiol predict mortality in elderly men. J Clin Endocrinol Metab.

[31] Schederecker F, Cecil A, Prehn C, Nano J, Koenig W, Adamski J, Zeller T, Peters A, Thorand B (2020). Sex hormone-binding globulin, androgens and mortality: the KORA-F4 cohort study. Endocr Connect.

[32] Benn M, Voss SS, Holmegard HN, Jensen GB, Tybjærg-Hansen A, Nordestgaard BG (2015). Extreme concentrations of endogenous sex hormones, ischemic heart disease, and death in women. Arterioscler Thromb Vasc Biol.

[33] Liu J, Zeng FF, Liu ZM, Zhang CX, Ling WH, Chen YM (2013). Effects of blood triglycerides on cardiovascular and all-cause mortality: a systematic review and meta-analysis of 61 prospective studies. Lipids Health Dis.

[34] Levine W, Dyer AR, Shekelle RB, Schoenberger JA, Stamler J (1989). Serum uric acid and 11.5-year mortality of middle-aged women: findings of the Chicago heart association detection project in industry. J Clin Epidemiol.

[35] Bai W, Li J, Liu J (2016). Serum phosphorus, cardiovascular and all-cause mortality in the general population: a meta-analysis. Clin Chim Acta.

[36] Zethelius B, Berglund L, Sundström J, Ingelsson E, Basu S, Larsson A, Venge P, Arnlöv J (2008). Use of multiple biomarkers to improve the prediction of death from cardiovascular causes. N Engl J Med.

[37] Coll E, Botey A, Alvarez L, Poch E, Quintó L, Saurina A, Vera M, Piera C, Darnell A (2000). Serum cystatin C as a new marker for noninvasive estimation of glomerular filtration rate and as a marker for early renal impairment. Am J Kidney Dis.

[38] Leto G, Crescimanno M, Flandina C (2018). On the role of cystatin C in cancer progression. Life Sci.

[39] Horvei LD, Grimnes G, Hindberg K, Mathiesen EB, Njolstad I, Wilsgaard T, Brox J, Braekkan SK, Hansen JB (2016). C-reactive protein, obesity, and the risk of arterial and venous thrombosis. J Thromb Haemost.

[40] Chomistek AK, Chiuve SE, Jensen MK, Cook NR, Rimm EB (2011). Vigorous physical activity, mediating biomarkers, and risk of myocardial infarction. Med Sci Sports Exerc.

[41] Shen Y, Zhou J, Shi L, Nauman E, Katzmarzyk PT, Price-Haywood EG, Horswell R, Bazzano AN, Nigam S, Hu G (2021). Association between visit-to-visit HbA1c variability and the risk of cardiovascular disease in patients with type 2 diabetes. Diabetes Obes Metab.

[42] Gao J, McCann A, Laupsa-Borge J, Nygård O, Ueland PM, Meyer K (2022). Within-person reproducibility of proteoforms related to inflammation and renal dysfunction. Sci Rep.

[43] Zhou J, Ge X, Fan X, Wang J, Miao L, Hang D (2021). Associations of vitamin D status with colorectal cancer risk and survival. Int J Cancer.

