# Supplementary material for: Development and validation of a blood biomarker score for predicting mortality risk in the general population
Source: J Transl Med. 2023 Jul 15;21:471. doi: 10.1186/s12967-023-04334-w (PMC10349520; doi:10.1186/s12967-023-04334-w)
Supplement: Supplementary file 1 — Additional file 1: Table S1. The assay methods and missingness of blood biomarkers (n = 451,170). [file 12967_2023_4334_MOESM1_ESM.docx]

| Table S1. The assay methods and missingness of blood biomarkers (n=451,170) | | | | | |
| --- | --- | --- | --- | --- | --- |
| Assay | Analytical platform | Men (n) | Missing proportion (%) | Women (n) | Missing proportion (%) |
| CRP | Beckman Coulter AU5800 | 188,568 | 6.37 | 232,757 | 6.81 |
| TC | Beckman Coulter AU5800 | 189,053 | 6.13 | 233,141 | 6.66 |
| TG | Beckman Coulter AU5800 | 188,849 | 6.23 | 233,018 | 6.71 |
| LDL-C | Beckman Coulter AU5800 | 188,641 | 6.33 | 232,767 | 6.81 |
| HDL-C | Beckman Coulter AU5800 | 174,618 | 13.29 | 211,915 | 15.16 |
| ApoA1 | Beckman Coulter AU5800 | 174,357 | 13.42 | 210,016 | 15.92 |
| ApoB | Beckman Coulter AU5800 | 187,754 | 6.77 | 232,347 | 6.98 |
| IGF-1 | DiaSorin Liaison XL | 188,058 | 6.62 | 231,853 | 7.18 |
| Testosterone | Beckman Coulter DXI 800 | 187,369 | 6.96 | 195,348 | 21.79 |
| FT | Beckman Coulter DXI 800 | 172,377 | 14.41 | 176,612 | 29.29 |
| SHBG | Beckman Coulter DXI 800 | 173,344 | 13.93 | 209,502 | 16.12 |
| HbA1c | Bio-Rad VARIANT II Turbo | 187,788 | 6.76 | 231,499 | 7.32 |
| Glucose | Beckman Coulter AU5800 | 174,502 | 13.35 | 211,752 | 15.22 |
| ALT | Beckman Coulter AU5800 | 188,896 | 6.21 | 233,136 | 6.66 |
| AST | Beckman Coulter AU5800 | 188,290 | 6.51 | 232,314 | 6.99 |
| GGT | Beckman Coulter AU5800 | 188,931 | 6.19 | 233,040 | 6.70 |
| ALP | Beckman Coulter AU5800 | 189,051 | 6.13 | 233,160 | 6.65 |
| TBIL | Beckman Coulter AU5800 | 188,231 | 6.54 | 232,160 | 7.05 |
| DBIL | Beckman Coulter AU5800 | 175,712 | 12.75 | 181,974 | 27.15 |
| TP | Beckman Coulter AU5800 | 174,508 | 13.35 | 211,792 | 15.21 |
| ALB | Beckman Coulter AU5800 | 174,722 | 13.24 | 211,991 | 15.13 |
| CysC | Siemens Advia 1800 | 189,026 | 6.14 | 233,145 | 6.66 |
| Creatinine | Beckman Coulter AU5800 | 188,947 | 6.18 | 233,038 | 6.70 |
| Urea | Beckman Coulter AU5800 | 188,916 | 6.20 | 232,990 | 6.72 |
| Urate | Beckman Coulter AU5800 | 188,843 | 6.23 | 232,861 | 6.77 |
| Calcium | Beckman Coulter AU5800 | 174,669 | 13.27 | 211,927 | 15.15 |
| Phosphate | Beckman Coulter AU5800 | 174,382 | 13.41 | 211,586 | 15.29 |
| 25(OH)D | DiaSorin Liaison XL | 182,787 | 9.24 | 219,804 | 12.00 |
| Abbreviations: CRP, C-reactive protein; TC, total cholesterol; TG, triglycerides; LDL-C, low-density lipoprotein cholesterol; HDL-C, high-density lipoprotein cholesterol; ApoA1, Apolipoprotein A1; ApoB, Apolipoprotein B; IGF-1, insulin‑like growth factor‑1; FT, free testosterone; SHBG, sex hormone-binding globulin; HbA1c, hemoglobin A1c; ALT, alanine aminotransferase; AST, aspartate aminotransferase; GGT, gamma-glutamyltransferase; ALP, alkaline phosphatase; TBIL, total bilirubin; DBIL, direct bilirubin; TP, total protein; ALB, albumin; CysC, cystatin C; 25(OH)D, 25-hydroxyvitamin D. | | | | | |
