# Supplementary material for: Development and validation of a blood biomarker score for predicting mortality risk in the general population
Source: J Transl Med. 2023 Jul 15;21:471. doi: 10.1186/s12967-023-04334-w (PMC10349520; doi:10.1186/s12967-023-04334-w)
Supplement: Supplementary file 2 — Additional file 2: Table S2. Comparison of baseline characteristics between the included and excluded UK Biobank participants. [file 12967_2023_4334_MOESM2_ESM.docx]

| **Table S1.** Comparison of baseline characteristics between the included and excluded UK Biobank participants | | | |
| --- | --- | --- | --- |
|  | Included (n=267,239) | Excluded (n=235,254) | SMD^b^ |
| Age in years, mean±SD | 55.8±8.2 | 57.4±7.9 | 0.19 |
| White race, n (%) | 253,167 (95) | 219,517 (94) | 0.04 |
| Townsend deprivation index, mean±SD | -1.40±3.04 | -1.17±3.16 | 0.07 |
| BMI in kg/m^2^, mean±SD | 27.3±4.7 | 27.6±4.9 | 0.05 |
| Physical activity in MET-hours/week, mean±SD | 44.9±45.8 | 43.2±44.6 | -0.04 |
| Smoking status, n (%)^a^ |  |  | 0.15 |
| Never | 148,780 (56) | 124,737 (53) |  |
| Previous | 90,795 (34) | 82,256 (35) |  |
| Current | 26,431 (10) | 26,546 (11) |  |
| Prevalent hypertension, n (%) | 69,420 (26) | 66,325 (28) | 0.15 |
| Prevalent diabetes, n (%) | 12,570 (5) | 13,829 (6) | 0.04 |
| Abbreviations: SD, standard deviation; BMI, body mass index; MET, metabolic equivalent; SMD, standardized mean difference. | | | |
| ^a^ The totals did not sum to 100% due to small proportions of participants choosing "prefer not to answer". | | | |
| ^b^ SMD > 0.2 indicates a potentially relevant difference between the groups. | | | |
