# Supplementary material for: Development and validation of a blood biomarker score for predicting mortality risk in the general population
Source: J Transl Med. 2023 Jul 15;21:471. doi: 10.1186/s12967-023-04334-w (PMC10349520; doi:10.1186/s12967-023-04334-w)
Supplement: Supplementary file 4 — Additional file 4: Table S4. Associations of blood biomarkers with all-cause mortality for men and women in the training set. [file 12967_2023_4334_MOESM4_ESM.docx]

| **Table S4.** Associations of blood biomarkers with all-cause mortality for men and women in the training set | | | | | |
| --- | --- | --- | --- | --- | --- |
|  |  | Men | | Women | |
|  |  | HR (95% CI)^a^ | *P* value | HR (95% CI)^a^ | *P* value |
| CRP | Age-adjusted model | 1.31 (1.28-1.34) | <0.0001 | 1.29 (1.25-1.33) | <0.0001 |
|  | Multivariable model^b^ | 1.24 (1.21-1.26) | <0.0001 | 1.19 (1.15-1.23) | <0.0001 |
| TC | Age-adjusted model | 0.85 (0.84-0.87) | <0.0001 | 0.86 (0.84-0.89) | <0.0001 |
|  | Multivariable model^b^ | 0.92 (0.90-0.94) | <0.0001 | 0.93 (0.90-0.96) | <0.0001 |
| TG | Age-adjusted model | 1.01 (0.99-1.03) | 0.48 | 1.13 (1.09-1.16) | <0.0001 |
|  | Multivariable model^b^ | 0.98 (0.96-1.00) | 0.07 | 1.02 (0.98-1.05) | 0.31 |
| LDL-C | Age-adjusted model | 0.85 (0.84-0.87) | <0.0001 | 0.89 (0.86-0.91) | <0.0001 |
|  | Multivariable model^b^ | 0.92 (0.90-0.93) | <0.0001 | 0.94 (0.91-0.97) | <0.0001 |
| HDL-C | Age-adjusted model | 0.91 (0.89-0.92) | <0.0001 | 0.84 (0.82-0.87) | <0.0001 |
|  | Multivariable model^b^ | 0.97 (0.95-0.99) | 0.02 | 0.94 (0.91-0.97) | <0.0001 |
| ApoA1 | Age-adjusted model | 0.90 (0.88-0.92) | <0.0001 | 0.87 (0.84-0.89) | <0.0001 |
|  | Multivariable model^b^ | 0.95 (0.93-0.97) | <0.0001 | 0.94 (0.91-0.97) | <0.0001 |
| ApoB | Age-adjusted model | 0.88 (0.87-0.90) | <0.0001 | 0.93 (0.90-0.96) | <0.0001 |
|  | Multivariable model^b^ | 0.93 (0.91-0.95) | <0.0001 | 0.96 (0.93-0.99) | 0.01 |
| IGF-1 | Age-adjusted model | 0.85 (0.84-0.87) | <0.0001 | 0.89 (0.86-0.92) | <0.0001 |
|  | Multivariable model^b^ | 0.89 (0.88-0.91) | <0.0001 | 0.93 (0.91-0.96) | <0.0001 |
| Testosterone | Age-adjusted model | 0.98 (0.96-1.00) | 0.08 | 1.09 (1.05-1.12) | <0.0001 |
|  | Multivariable model^b^ | 0.92 (0.90-0.94) | <0.0001 | 1.05 (1.02-1.08) | 0.0008 |
| FT | Age-adjusted model | 0.88 (0.86-0.90) | <0.0001 | 1.08 (1.05-1.12) | <0.0001 |
|  | Multivariable model^b^ | 0.90 (0.89-0.92) | <0.0001 | 0.99 (0.96-1.03) | 0.81 |
| SHBG | Age-adjusted model | 1.13 (1.11-1.16) | <0.0001 | 0.98 (0.95-1.01) | 0.98 |
|  | Multivariable model^b^ | 1.20 (1.17-1.23) | <0.0001 | 1.10 (1.07-1.14) | <0.0001 |
| HbA1c | Age-adjusted model | 1.21 (1.19-1.23) | <0.0001 | 1.18 (1.15-1.21) | <0.0001 |
|  | Multivariable model^b^ | 1.10 (1.07-1.12) | <0.0001 | 1.03 (0.99-1.07) | 0.06 |
| Glucose | Age-adjusted model | 1.15 (1.13-1.17) | <0.0001 | 1.12 (1.09-1.15) | <0.0001 |
|  | Multivariable model^b^ | 1.07 (1.05-1.09) | <0.0001 | 1.04 (1.01-1.07) | 0.01 |
| ALT | Age-adjusted model | 0.99 (0.98-1.02) | 0.88 | 1.05 (1.02-1.09) | 0.0007 |
|  | Multivariable model^b^ | 0.99 (0.97-1.01) | 0.35 | 0.99 (0.97-1.03) | 0.78 |
| AST | Age-adjusted model | 1.10 (1.07-1.12) | <0.0001 | 1.10 (1.07-1.14) | <0.0001 |
|  | Multivariable model^b^ | 1.09 (1.07-1.11) | <0.0001 | 1.09 (1.06-1.12) | <0.0001 |
| GGT | Age-adjusted model | 1.26 (1.24-1.29) | <0.0001 | 1.20 (1.17-1.23) | <0.0001 |
|  | Multivariable model^b^ | 1.20 (1.18-1.23) | <0.0001 | 1.12 (1.09-1.16) | <0.0001 |
| ALP | Age-adjusted model | 1.22 (1.19-1.24) | <0.0001 | 1.16 (1.12-1.20) | <0.0001 |
|  | Multivariable model^b^ | 1.17 (1.15-1.20) | <0.0001 | 1.09 (1.06-1.13) | <0.0001 |
| TBIL | Age-adjusted model | 0.91 (0.89-0.93) | <0.0001 | 0.94 (0.91-0.97) | 0.0003 |
|  | Multivariable model^b^ | 0.96 (0.94-0.98) | 0.0006 | 0.99 (0.96-1.02) | 0.47 |
| DBIL | Age-adjusted model | 1.02 (0.99-1.04) | 0.13 | 1.04 (1.00-1.07) | 0.03 |
|  | Multivariable model^b^ | 1.04 (1.02-1.06) | 0.0002 | 1.06 (1.02-1.09) | 0.0006 |
| TP | Age-adjusted model | 1.05 (1.03-1.07) | <0.0001 | 1.05 (1.02-1.08) | 0.0022 |
|  | Multivariable model^b^ | 1.04 (1.02-1.06) | <0.0001 | 1.06 (1.03-1.10) | 0.0002 |
| ALB | Age-adjusted model | 0.82 (0.80-0.84) | <0.0001 | 0.86 (0.83-0.88) | <0.0001 |
|  | Multivariable model^b^ | 0.83 (0.81-0.85) | <0.0001 | 0.89 (0.86-0.92) | <0.0001 |
| CysC | Age-adjusted model | 1.31 (1.29-1.34) | <0.0001 | 1.37 (1.34-1.41) | <0.0001 |
|  | Multivariable model^b^ | 1.24 (1.22-1.26) | <0.0001 | 1.27 (1.24-1.31) | <0.0001 |
| Creatinine | Age-adjusted model | 0.96 (0.94-0.97) | <0.0001 | 1.03 (0.99-1.06) | 0.06 |
|  | Multivariable model^b^ | 0.98 (0.96-0.99) | 0.02 | 1.02 (0.99-1.05) | 0.18 |
| Urea | Age-adjusted model | 0.90 (0.88-0.92) | <0.0001 | 0.99 (0.97-1.03) | 0.94 |
|  | Multivariable model^b^ | 0.92 (0.90-0.94) | <0.0001 | 0.99 (0.96-1.02) | 0.49 |
| Urate | Age-adjusted model | 1.02 (0.99-1.04) | 0.06 | 1.18 (1.14-1.21) | <0.0001 |
|  | Multivariable model^b^ | 0.99 (0.98-1.02) | 0.77 | 1.07 (1.03-1.10) | 0.0001 |
| Calcium | Age-adjusted model | 1.06 (1.04-1.08) | <0.0001 | 1.05 (1.02-1.08) | 0.0007 |
|  | Multivariable model^b^ | 1.03 (1.01-1.05) | 0.01 | 1.02 (0.99-1.05) | 0.24 |
| Phosphate | Age-adjusted model | 1.03 (1.01-1.06) | 0.0017 | 0.97 (0.94-1.00) | 0.06 |
|  | Multivariable model^b^ | 1.02 (0.99-1.04) | 0.12 | 0.98 (0.95-1.01) | 0.24 |
| 25(OH)D | Age-adjusted model | 0.80 (0.78-0.81) | <0.0001 | 0.82 (0.79-0.84) | <0.0001 |
|  | Multivariable model^b^ | 0.84 (0.83-0.86) | <0.0001 | 0.87 (0.85-0.90) | <0.0001 |
| Abbreviations: HR, hazard ratio; CI, confidence interval; CRP, C-reactive protein; TC, total cholesterol; TG, triglycerides; LDL-C, low-density lipoprotein cholesterol; HDL-C, high-density lipoprotein cholesterol; ApoA1, Apolipoprotein A1; ApoB, Apolipoprotein B; IGF-1, insulin‑like growth factor‑1; FT, free testosterone; SHBG, sex hormone-binding globulin; HbA1c, hemoglobin A1c; ALT, alanine aminotransferase; AST, aspartate aminotransferase; GGT, gamma-glutamyltransferase; ALP, alkaline phosphatase; TBIL, total bilirubin; DBIL, direct bilirubin; TP, total protein; ALB, albumin; CysC, cystatin C; 25(OH)D, 25-hydroxyvitamin D. | | | | | |
| ^a^ HR per 1-SD increase in log-transformed biomarker concentration. | | | | | |
| ^b^ Adjusted for age, BMI, physical activity, smoking status, prevalent hypertension, prevalent diabetes, and additionally adjusted for SHBG when evaluating the association between testosterone and mortality. | | | | | |
