# Supplementary material for: Development and validation of a blood biomarker score for predicting mortality risk in the general population
Source: J Transl Med. 2023 Jul 15;21:471. doi: 10.1186/s12967-023-04334-w (PMC10349520; doi:10.1186/s12967-023-04334-w)
Supplement: Supplementary file 5 — Additional file 5: Table S5. Mediation effects of predictive biomarkers for the associations between traditional risk factors and all-cause mortality in men. [file 12967_2023_4334_MOESM5_ESM.docx]

| **Table S5**. Mediation effects of predictive biomarkers for the associations between traditional risk factors and all-cause mortality in men | | | | | | | | | | | | | | |
| --- | --- | --- | --- | --- | --- | --- | --- | --- | --- | --- | --- | --- | --- | --- |
| Traditional risk factor^a^ | Score | CRP | LDL-C | IGF-1 | FT | SHBG | HbA1c | Glucose | GGT | ALP | ALB | CysC | Creatinine | 25(OH)D |
| **Current smoking** | | | | | | | | | | | | | | |
| Total effect | 2.22 (2.12-2.34) | 2.22 (2.12-2.34) | 2.22 (2.12-2.34) | 2.22 (2.12-2.34) | 2.22 (2.12-2.34) | 2.22 (2.12-2.34) | 2.22 (2.12-2.34) | 2.22 (2.12-2.34) | 2.22 (2.12-2.34) | 2.22 (2.12-2.34) | 2.22 (2.12-2.34) | 2.22 (2.12-2.34) | 2.22 (2.12-2.34) | 2.22 (2.12-2.34) |
| Direct effect | 1.68 (1.60-1.77) | 2.06 (1.96-2.17) | 2.22 (2.11-2.33) | 2.19 (2.08-2.30) | 2.21 (2.10-2.32) | 2.17 (2.07-2.28) | 2.15 (2.04-2.26) | 2.25 (2.14-2.36) | 2.17 (2.07-2.28) | 2.13 (2.03-2.24) | 2.17 (2.07-2.28) | 2.04 (1.94-2.14) | 2.21 (2.11-2.33) | 2.10 (2.00-2.21) |
| Proportion mediated % | 34.9% (32.1%-37.8%) | 9.4% (8.3%-10.6%) | Null | 2.0% (1.5%-2.6%) | Null | 2.9% (2.2%-3.7%) | 4.3% (3.6%-5.1%) | Null | 2.8% (2.2%-3.7%) | 5.5% (4.7%-6.4%) | 3.0% (2.4%-3.7%) | 10.8% (9.6%-12.2%) | Null | 7.2% (6.2%-8.3%) |
| *P* value | <0.0001 | <0.0001 | - | <0.0001 | - | <0.0001 | <0.0001 | - | <0.0001 | <0.0001 | <0.0001 | <0.0001 | - | <0.0001 |
| **Obesity** | | | | | | | | | | | | | | |
| Total effect | 1.32 (1.26-1.37) | 1.32 (1.26-1.37) | 1.32 (1.26-1.37) | 1.32 (1.26-1.37) | 1.32 (1.26-1.37) | 1.32 (1.26-1.37) | 1.32 (1.26-1.37) | 1.32 (1.26-1.37) | 1.32 (1.26-1.37) | 1.32 (1.26-1.37) | 1.32 (1.26-1.37) | 1.32 (1.26-1.37) | 1.32 (1.26-1.37) | 1.32 (1.26-1.37) |
| Direct effect | 1.00 (0.96-1.05) | 1.16 (1.11-1.21) | 1.28 (1.23-1.33) | 1.26 (1.21-1.32) | 1.29 (1.23-1.34) | 1.42 (1.36-1.48) | 1.21 (1.15-1.26) | 1.26 (1.21-1.31) | 1.20 (1.14-1.25) | 1.30 (1.24-1.35) | 1.29 (1.24-1.35) | 1.17 (1.12-1.22) | 1.32 (1.27-1.38) | 1.25 (1.20-1.31) |
| Proportion mediated % | 99.1% (0.0%-100.0%) | 46.2% (38.2%-54.3%) | 10.3% (8.2%-12.9%) | 15.4% (12.1%-19.4%) | 7.6% (5.9%-9.7%) | Null | 31.9% (26.0%-38.4%) | 15.9% (12.5%-19.9%) | 34.9% (28.7%-41.6%) | 4.8% (3.5%-6.4%) | 6.6% (5.1%-8.4%) | 42.1% (34.9%-49.6%) | Null | 18.3% (15.0%-22.1%) |
| *P* value | <0.0001 | <0.0001 | <0.0001 | <0.0001 | <0.0001 | - | <0.0001 | <0.0001 | <0.0001 | <0.0001 | <0.0001 | <0.0001 | - | <0.0001 |
| **Physical inactivity** | | | | | | | | | | | | | | |
| Total effect | 1.10 (1.06-1.14) | 1.10 (1.06-1.14) | 1.10 (1.06-1.14) | 1.10 (1.06-1.14) | 1.10 (1.06-1.14) | 1.10 (1.06-1.14) | 1.10 (1.06-1.14) | 1.10 (1.06-1.14) | 1.10 (1.06-1.14) | 1.10 (1.06-1.14) | 1.10 (1.06-1.14) | 1.10 (1.06-1.14) | 1.10 (1.06-1.14) | 1.10 (1.06-1.14) |
| Direct effect | 1.05 (1.01-1.09) | 1.07 (1.03-1.12) | 1.09 (1.05-1.14) | 1.10 (1.06-1.14) | 1.10 (1.06-1.14) | 1.12 (1.07-1.16) | 1.08 (1.04-1.13) | 1.09 (1.05-1.13) | 1.07 (1.03-1.11) | 1.10 (1.06-1.14) | 1.10 (1.05-1.14) | 1.07 (1.03-1.11) | 1.10 (1.06-1.14) | 1.04 (1.00-1.09) |
| Proportion mediated % | 45.9% (26.8%-66.4%) | 22.7% (14.0%-34.6%) | 6.3% (3.5%-11.1%) | Null | Null | Null | 13.5% (8.1%-21.6%) | 8.4% (4.9%-14.1%) | 25.9% (16.0%-39.0%) | Null | 2.6% (0.8%-8.1%) | 29.6% (18.1%-44.4%) | Null | 54.1% (31.4%-75.2%) |
| *P* value | <0.0001 | <0.0001 | <0.0001 | - | - | - | <0.0001 | <0.0001 | <0.0001 | - | 0.0353 | <0.0001 | - | <0.0001 |
| **Prevalent hypertension** | | | | | | | | | | | | | | |
| Total effect | 1.30 (1.25-1.35) | 1.30 (1.25-1.35) | 1.30 (1.25-1.35) | 1.30 (1.25-1.35) | 1.30 (1.25-1.35) | 1.30 (1.25-1.35) | 1.30 (1.25-1.35) | 1.30 (1.25-1.35) | 1.30 (1.25-1.35) | 1.30 (1.25-1.35) | 1.30 (1.25-1.35) | 1.30 (1.25-1.35) | 1.30 (1.25-1.35) | 1.30 (1.25-1.35) |
| Direct effect | 1.10 (1.05-1.14) | 1.24 (1.19-1.29) | 1.22 (1.17-1.27) | 1.29 (1.24-1.34) | 1.29 (1.23-1.34) | 1.35 (1.29-1.40) | 1.23 (1.18-1.28) | 1.25 (1.20-1.30) | 1.22 (1.17-1.27) | 1.28 (1.23-1.34) | 1.34 (1.28-1.39) | 1.19 (1.15-1.24) | 1.31 (1.26-1.36) | 1.28 (1.23-1.33) |
| Proportion mediated % | 65.2% (53.6%-75.2%) | 18.2% (15.1%-21.8%) | 24.3% (19.3%-30.0%) | 2.7% (1.8%-4.0%) | 4.1% (3.1%-5.4%) | Null | 21.6% (17.7%-26.2%) | 14.2% (11.3%-17.7%) | 25.2% (20.9%-30.1%) | 5.1% (3.8%-6.7%) | Null | 32.1% (26.8%-38.2%) | Null | 6.2% (4.7%-8.2%) |
| *P* value | <0.0001 | <0.0001 | <0.0001 | <0.0001 | <0.0001 | - | <0.0001 | <0.0001 | <0.0001 | <0.0001 | - | <0.0001 | - | <0.0001 |
| **Prevalent diabetes** | | | | | | | | | | | | | | |
| Total effect | 1.81 (1.71-1.92) | 1.81 (1.71-1.92) | 1.81 (1.71-1.92) | 1.81 (1.71-1.92) | 1.81 (1.71-1.92) | 1.81 (1.71-1.92) | 1.81 (1.71-1.92) | 1.81 (1.71-1.92) | 1.81 (1.71-1.92) | 1.81 (1.71-1.92) | 1.81 (1.71-1.92) | 1.81 (1.71-1.92) | 1.81 (1.71-1.92) | 1.81 (1.71-1.92) |
| Direct effect | 1.13 (1.06-1.21) | 1.78 (1.68-1.89) | 1.59 (1.49-1.70) | 1.74 (1.64-1.85) | 1.77 (1.66-1.87) | 1.92 (1.81-2.04) | 1.34 (1.24-1.45) | 1.59 (1.48-1.71) | 1.71 (1.61-1.82) | 1.80 (1.69-1.91) | 1.84 (1.73-1.95) | 1.70 (1.60-1.80) | 1.80 (1.70-1.91) | 1.73 (1.63-1.84) |
| Proportion mediated % | 78.8% (68.5%-86.4%) | 2.5% (1.5%-4.1%) | 21.8% (17.2%-27.2%) | 6.4% (5.1%-8.1%) | 4.2% (3.3%-5.3%) | Null | 50.9% (40.9%-60.9%) | 21.5% (15.4%-29.3%) | 9.4% (7.8%-11.3%) | 1.4% (0.7%-2.8%) | Null | 11.0% (8.9%-13.6%) | Null | 7.3% (5.9%-9.0%) |
| *P* value | <0.0001 | <0.0001 | <0.0001 | <0.0001 | <0.0001 | - | <0.0001 | <0.0001 | <0.0001 | 0.0023 | - | <0.0001 | - | <0.0001 |
| Abbreviations: Null, not mediating the effect; CRP, C-reactive protein; LDL-C, low-density lipoprotein cholesterol; IGF-1, insulin‑like growth factor‑1; FT, free testosterone; SHBG, sex hormone-binding globulin; HbA1c, hemoglobin A1c; GGT, gamma-glutamyltransferase; ALP, alkaline phosphatase; ALB, albumin; CysC, cystatin C; 25(OH)D, 25-hydroxyvitamin D. | | | | | | | | | | | | | | |
| Hazard ratios and corresponding 95% confidence intervals are provided for the total and direct effects. Models were adjusted for age, ethnicity, and Townsend deprivation index. | | | | | | | | | | | | | | |
| ^a^ Traditional risk factors were entered as dichotomous variables. The reference group was non-current smoking, non-obesity (BMI<30kg/m^2^), physically active (MET-hours/week>median value), non-prevalent hypertension, and non-prevalent diabetes, respectively. | | | | | | | | | | | | | | |
