# Supplementary material for: Development and validation of a blood biomarker score for predicting mortality risk in the general population
Source: J Transl Med. 2023 Jul 15;21:471. doi: 10.1186/s12967-023-04334-w (PMC10349520; doi:10.1186/s12967-023-04334-w)
Supplement: Supplementary file 6 — Additional file 6: Table S6. Mediation effects of predictive biomarkers for the associations between traditional risk factors and all-cause mortality in women. [file 12967_2023_4334_MOESM6_ESM.docx]

| **Table S6**. Mediation effects of predictive biomarkers for the associations between traditional risk factors and all-cause mortality in women | | | | | | | | | | | | | | | | | | |
| --- | --- | --- | --- | --- | --- | --- | --- | --- | --- | --- | --- | --- | --- | --- | --- | --- | --- | --- |
| Traditional risk factor^a^ | Score | CRP | TG | LDL-C | ApoA1 | IGF-1 | Testosterone | FT | HbA1c | Glucose | ALT | AST | GGT | ALP | ALB | CysC | Calcium | 25(OH)D |
| **Current smoking** | | | | | | | | | | | | | | | | | | |
| Total effect | 2.54 (2.35-2.75) | 2.54 (2.35-2.75) | 2.54 (2.35-2.75) | 2.54 (2.35-2.75) | 2.54 (2.35-2.75) | 2.54 (2.35-2.75) | 2.54 (2.35-2.75) | 2.54 (2.35-2.75) | 2.54 (2.35-2.75) | 2.54 (2.35-2.75) | 2.54 (2.35-2.75) | 2.54 (2.35-2.75) | 2.54 (2.35-2.75) | 2.54 (2.35-2.75) | 2.54 (2.35-2.75) | 2.54 (2.35-2.75) | 2.54 (2.35-2.75) | 2.54 (2.35-2.75) |
| Direct effect | 2.26 (2.08-2.44) | 2.45 (2.26-2.65) | 2.49 (2.30-2.70) | 2.54 (2.35-2.75) | 2.49 (2.30-2.70) | 2.53 (2.34-2.74) | 2.52 (2.33-2.73) | 2.54 (2.35-2.75) | 2.46 (2.27-2.66) | 2.56 (2.36-2.77) | 2.57 (2.37-2.78) | 2.59 (2.40-2.81) | 2.48 (2.29-2.68) | 2.50 (2.31-2.71) | 2.52 (2.33-2.73) | 2.28 (2.11-2.47) | 2.53 (2.34-2.74) | 2.45 (2.26-2.65) |
| Proportion mediated % | 12.8% (11.1%-14.7%) | 3.9% (3.1%-5.0%) | 2.1% (1.5%-3.0%) | Null | 2.0% (1.4%-2.8%) | Null | Null | Null | 3.4% (2.7%-4.4%) | Null | Null | Null | 2.6% (2.0%-3.4%) | 1.7% (1.2%-2.3%) | Null | 11.4% (9.8%-13.2%) | Null | 4.1% (3.2%-5.2%) |
| *P* value | <0.0001 | <0.0001 | <0.0001 | - | <0.0001 | - | - | - | <0.0001 | - | - | - | <0.0001 | <0.0001 | - | <0.0001 | - | <0.0001 |
| **Obesity** | | | | | | | | | | | | | | | | | | |
| Total effect | 1.38 (1.30-1.47) | 1.38 (1.30-1.47) | 1.38 (1.30-1.47) | 1.38 (1.30-1.47) | 1.38 (1.30-1.47) | 1.38 (1.30-1.47) | 1.38 (1.30-1.47) | 1.38 (1.30-1.47) | 1.38 (1.30-1.47) | 1.38 (1.30-1.47) | 1.38 (1.30-1.47) | 1.38 (1.30-1.47) | 1.38 (1.30-1.47) | 1.38 (1.30-1.47) | 1.38 (1.30-1.47) | 1.38 (1.30-1.47) | 1.38 (1.30-1.47) | 1.38 (1.30-1.47) |
| Direct effect | 0.95 (0.88-1.01) | 1.15 (1.08-1.23) | 1.32 (1.24-1.41) | 1.37 (1.29-1.46) | 1.31 (1.23-1.39) | 1.35 (1.26-1.43) | 1.36 (1.28-1.45) | 1.35 (1.27-1.44) | 1.29 (1.21-1.38) | 1.34 (1.26-1.43) | 1.37 (1.28-1.46) | 1.36 (1.28-1.45) | 1.27 (1.19-1.36) | 1.33 (1.25-1.42) | 1.33 (1.25-1.42) | 1.14 (1.07-1.21) | 1.38 (1.29-1.46) | 1.29 (1.21-1.38) |
| Proportion mediated % | 100% | 56.4% (42.7%-69.2%) | 14.3% (9.2%-21.6%) | 2.3% (1.5%-3.6%) | 17.2% (12.1%-23.8%) | 8.5% (5.3%-13.3%) | 4.0% (2.5%-6.5%) | 6.4% (2.5%-15.6%) | 20.7% (15.2%-27.5%) | 9.4% (6.5%-13.5%) | Null | 4.3% (2.8%-6.7%) | 25.6% (19.7%-32.7%) | 11.3% (8.0%-15.8%) | 11.4% (8.0%-15.9%) | 60.1% (46.9%-71.9%) | 1.5% (0.8%-3.0%) | 21.1% (16.1%-27.3%) |
| *P* value | <0.0001 | <0.0001 | <0.0001 | <0.0001 | <0.0001 | <0.0001 | <0.0001 | 0.0156 | <0.0001 | <0.0001 | - | <0.0001 | <0.0001 | <0.0001 | <0.0001 | <0.0001 | 0.0014 | <0.0001 |
| **Physical inactivity** | | | | | | | | | | | | | | | | | | |
| Total effect | 1.18 (1.11-1.25) | 1.18 (1.11-1.25) | 1.18 (1.11-1.25) | 1.18 (1.11-1.25) | 1.18 (1.11-1.25) | 1.18 (1.11-1.25) | 1.18 (1.11-1.25) | 1.18 (1.11-1.25) | 1.18 (1.11-1.25) | 1.18 (1.11-1.25) | 1.18 (1.11-1.25) | 1.18 (1.11-1.25) | 1.18 (1.11-1.25) | 1.18 (1.11-1.25) | 1.18 (1.11-1.25) | 1.18 (1.11-1.25) | 1.18 (1.11-1.25) | 1.18 (1.11-1.25) |
| Direct effect | 1.08 (1.01-1.14) | 1.12 (1.06-1.19) | 1.16 (1.09-1.23) | 1.17 (1.11-1.25) | 1.15 (1.09-1.22) | 1.17 (1.10-1.24) | 1.17 (1.10-1.24) | 1.17 (1.10-1.24) | 1.16 (1.09-1.23) | 1.17 (1.10-1.24) | 1.17 (1.10-1.24) | 1.18 (1.11-1.25) | 1.15 (1.08-1.22) | 1.16 (1.10-1.23) | 1.17 (1.10-1.24) | 1.12 (1.05-1.18) | 1.18 (1.11-1.25) | 1.14 (1.07-1.21) |
| Proportion mediated % | 54.1% (34.1%-72.9%) | 29.8% (19.6%-42.5%) | 9.0% (5.6%-14.2%) | 1.6% (0.7%-3.5%) | 12.0% (7.6%-18.4%) | 3.0% (1.7%-5.3%) | 2.9% (1.6%-5.2%) | 6.0% (3.3%-10.6%) | 8.4% (5.2%-13.1%) | 3.4% (1.9%-6.1%) | 2.7% (1.3%-5.6%) | Null | 16.0% (10.5%-23.7%) | 7.4% (4.6%-11.7%) | 5.3% (3.2%-8.5%) | 32.6% (21.5%-46.2%) | Null | 20.8% (13.7%-30.5%) |
| *P* value | <0.0001 | <0.0001 | <0.0001 | 0.0038 | <0.0001 | <0.0001 | <0.0001 | <0.0001 | <0.0001 | <0.0001 | 0.0008 | - | <0.0001 | <0.0001 | <0.0001 | <0.0001 | - | <0.0001 |
| **Prevalent hypertension** | | | | | | | | | | | | | | | | | | |
| Total effect | 1.36 (1.28-1.45) | 1.36 (1.28-1.45) | 1.36 (1.28-1.45) | 1.36 (1.28-1.45) | 1.36 (1.28-1.45) | 1.36 (1.28-1.45) | 1.36 (1.28-1.45) | 1.36 (1.28-1.45) | 1.36 (1.28-1.45) | 1.36 (1.28-1.45) | 1.36 (1.28-1.45) | 1.36 (1.28-1.45) | 1.36 (1.28-1.45) | 1.36 (1.28-1.45) | 1.36 (1.28-1.45) | 1.36 (1.28-1.45) | 1.36 (1.28-1.45) | 1.36 (1.28-1.45) |
| Direct effect | 1.13 (1.06-1.20) | 1.27 (1.19-1.35) | 1.32 (1.24-1.41) | 1.32 (1.24-1.40) | 1.33 (1.25-1.41) | 1.36 (1.28-1.44) | 1.35 (1.27-1.44) | 1.34 (1.26-1.42) | 1.30 (1.22-1.38) | 1.33 (1.25-1.41) | 1.35 (1.27-1.43) | 1.34 (1.26-1.42) | 1.29 (1.21-1.37) | 1.34 (1.26-1.42) | 1.37 (1.29-1.46) | 1.22 (1.14-1.29) | 1.35 (1.27-1.43) | 1.34 (1.26-1.42) |
| Proportion mediated % | 60.8% (47.4%-72.7%) | 23.2% (18.1%-29.2%) | 9.0% (6.2%-13.0%) | 9.6% (6.5%-13.9%) | 7.7% (5.5%-10.6%) | 1.3% (0.7%-2.4%) | 2.2% (1.4%-3.6%) | 5.0% (2.7%-8.8%) | 15.3% (11.3%-20.5%) | 8.6% (5.9%-12.3%) | 3.3% (1.4%-7.6%) | 4.7% (3.1%-7.3%) | 17.8% (13.7%-22.8%) | 5.7% (4.1%-7.9%) | Null | 36.3% (28.5%-44.8%) | 2.5% (1.2%-5.2%) | 5.6% (4.1%-7.6%) |
| *P* value | <0.0001 | <0.0001 | <0.0001 | <0.0001 | <0.0001 | 0.0002 | <0.0001 | 0.0002 | <0.0001 | <0.0001 | 0.0087 | <0.0001 | <0.0001 | <0.0001 | - | <0.0001 | 0.0032 | <0.0001 |
| **Prevalent diabetes** | | | | | | | | | | | | | | | | | | |
| Total effect | 2.09 (1.88-2.32) | 2.09 (1.88-2.32) | 2.09 (1.88-2.32) | 2.09 (1.88-2.32) | 2.09 (1.88-2.32) | 2.09 (1.88-2.32) | 2.09 (1.88-2.32) | 2.09 (1.88-2.32) | 2.09 (1.88-2.32) | 2.09 (1.88-2.32) | 2.09 (1.88-2.32) | 2.09 (1.88-2.32) | 2.09 (1.88-2.32) | 2.09 (1.88-2.32) | 2.09 (1.88-2.32) | 2.09 (1.88-2.32) | 2.09 (1.88-2.32) | 2.09 (1.88-2.32) |
| Direct effect | 1.32 (1.18-1.48) | 1.93 (1.74-2.14) | 2.00 (1.80-2.22) | 1.92 (1.72-2.15) | 1.96 (1.77-2.18) | 2.04 (1.84-2.27) | 2.09 (1.88-2.31) | 2.04 (1.84-2.27) | 1.67 (1.46-1.90) | 1.90 (1.69-2.13) | 2.05 (1.85-2.28) | 2.06 (1.86-2.29) | 1.92 (1.73-2.13) | 2.04 (1.83-2.26) | 2.06 (1.86-2.29) | 1.81 (1.63-2.01) | 2.07 (1.86-2.29) | 1.98 (1.78-2.19) |
| Proportion mediated % | 62.0% (51.3%-71.7%) | 10.9% (8.8%-13.5%) | 5.7% (4.0%-8.1%) | 11.3% (6.8%-18.1%) | 8.3% (6.1%-11.3%) | 3.0% (1.9%-4.6%) | Null | 3.1% (1.8%-5.5%) | 30.7% (19.9%-44.2%) | 12.9% (6.9%-22.7%) | 2.3% (0.9%-5.4%) | 1.6% (0.9%-2.9%) | 11.2% (8.9%-14.1%) | 3.5% (2.5%-4.9%) | 1.6% (0.9%-2.8%) | 19.5% (15.5%-24.2%) | 1.3% (0.7%-2.7%) | 7.5% (5.8%-9.7%) |
| *P* value | <0.0001 | <0.0001 | <0.0001 | <0.0001 | <0.0001 | <0.0001 | - | 0.0002 | <0.0001 | 0.0004 | 0.0118 | 0.0003 | <0.0001 | <0.0001 | 0.0001 | <0.0001 | 0.0024 | <0.0001 |
| Abbreviations: Null, not mediating the effect; CRP, C-reactive protein; TG, triglycerides; LDL-C, low-density lipoprotein cholesterol; ApoA1, Apolipoprotein A1; IGF-1, insulin‑like growth factor‑1; FT, free testosterone; HbA1c, hemoglobin A1c; ALT, alanine aminotransferase; AST, aspartate aminotransferase; GGT, gamma-glutamyltransferase; ALP, alkaline phosphatase; ALB, albumin; CysC, cystatin C; 25(OH)D, 25-hydroxyvitamin D. | | | | | | | | | | | | | | | | | | |
| Hazard ratios and corresponding 95% confidence intervals are provided for the total and direct effects. Models were adjusted for age, ethnicity, and Townsend deprivation index. | | | | | | | | | | | | | | | | | | |
| ^a^ Traditional risk factors were entered as dichotomous variables. The reference group was non-current smoking, non-obesity (BMI<30kg/m^2^), physically active (MET-hours/week>median value), non-prevalent hypertension, and non-prevalent diabetes, respectively. | | | | | | | | | | | | | | | | | | |
