# Supplementary figures and images for: Development and validation of a blood biomarker score for predicting mortality risk in the general population
Source: J Transl Med. 2023 Jul 15;21:471. doi: 10.1186/s12967-023-04334-w (PMC10349520; doi:10.1186/s12967-023-04334-w)

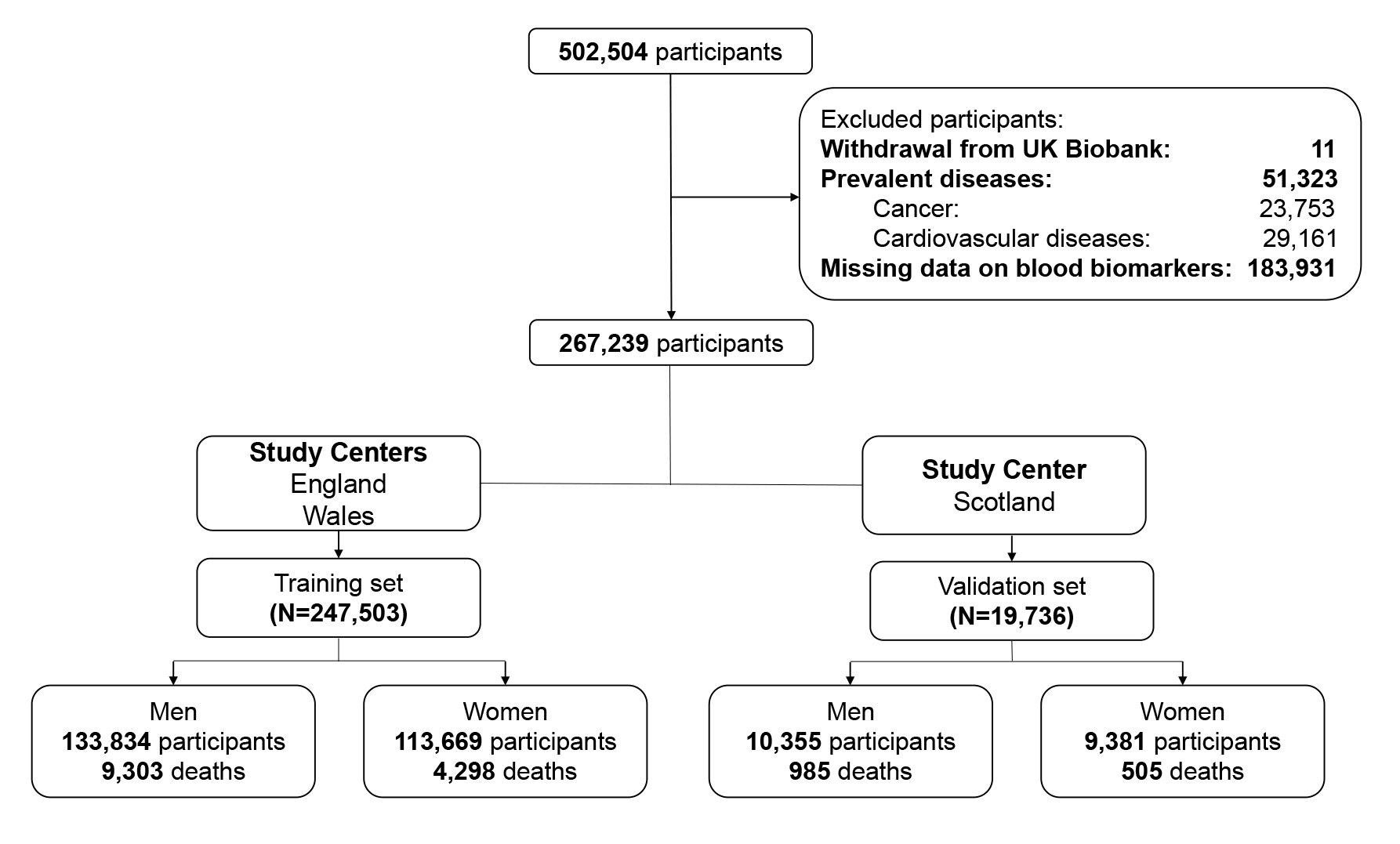

Supplement: Supplementary file 7 — Additional file 7: Figure S1. Flowchart of study population selection. [file 12967_2023_4334_MOESM7_ESM.tif]

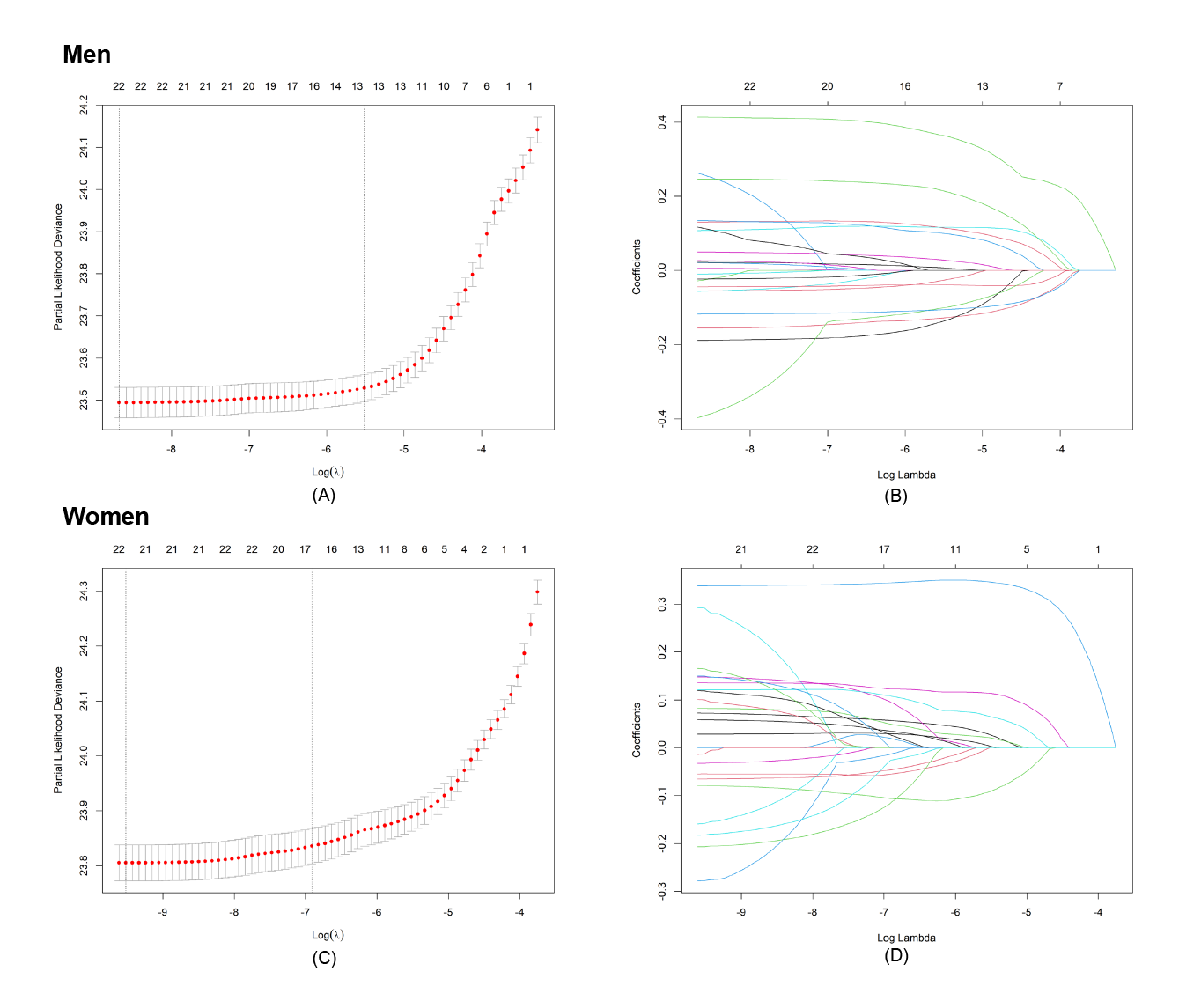

Supplement: Supplementary file 8 — Additional file 8: Figure S2. Biomarker selection using the Least absolute shrinkage and selection operator (LASSO) regression in the training set for men and women. Ten-fold cross-validation for tuning parameter selection in the LASSO regression (A and C); LASSO coefficient profiles of the 28 candidate biomarkers (B and D). [file 12967_2023_4334_MOESM8_ESM.tif]

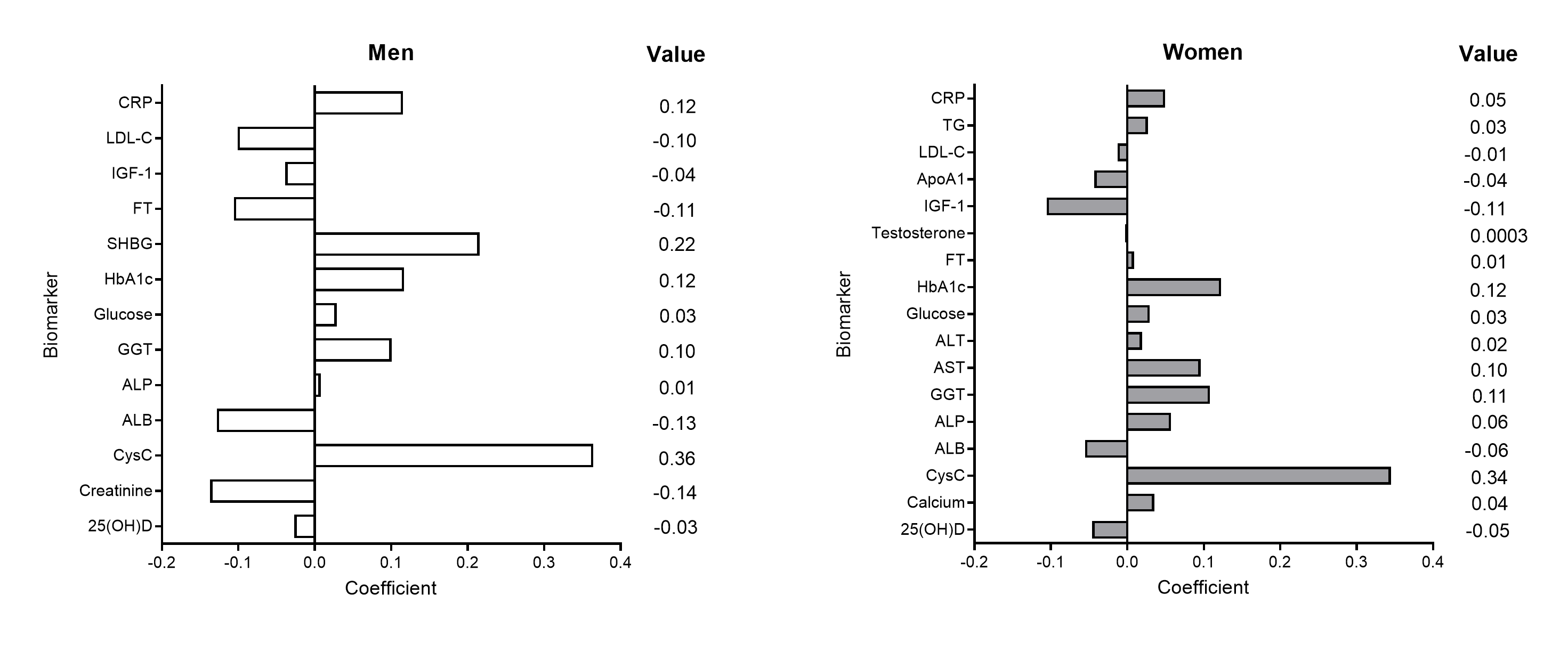

Supplement: Supplementary file 9 — Additional file 9: Figure S3. The coefficients of selected biomarkers derived from the LASSO regression for all-cause mortality in the training set. [file 12967_2023_4334_MOESM9_ESM.tif]

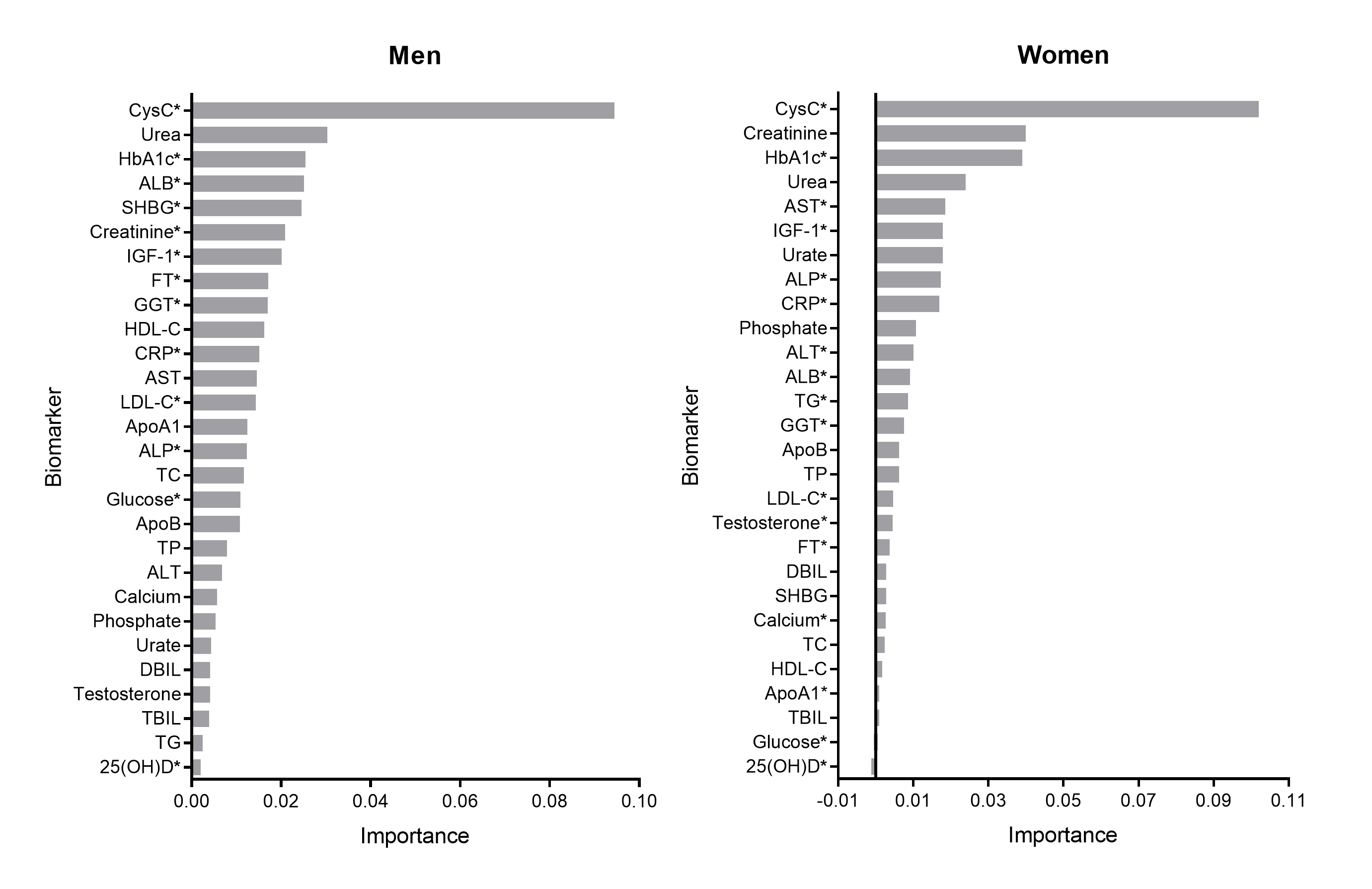

Supplement: Supplementary file 10 — Additional file 10: Figure S4. Variable importance based on random survival forest models for all-cause mortality prediction. The asterisk denotes the variables selected from the LASSO regression in the training set. [file 12967_2023_4334_MOESM10_ESM.tif]

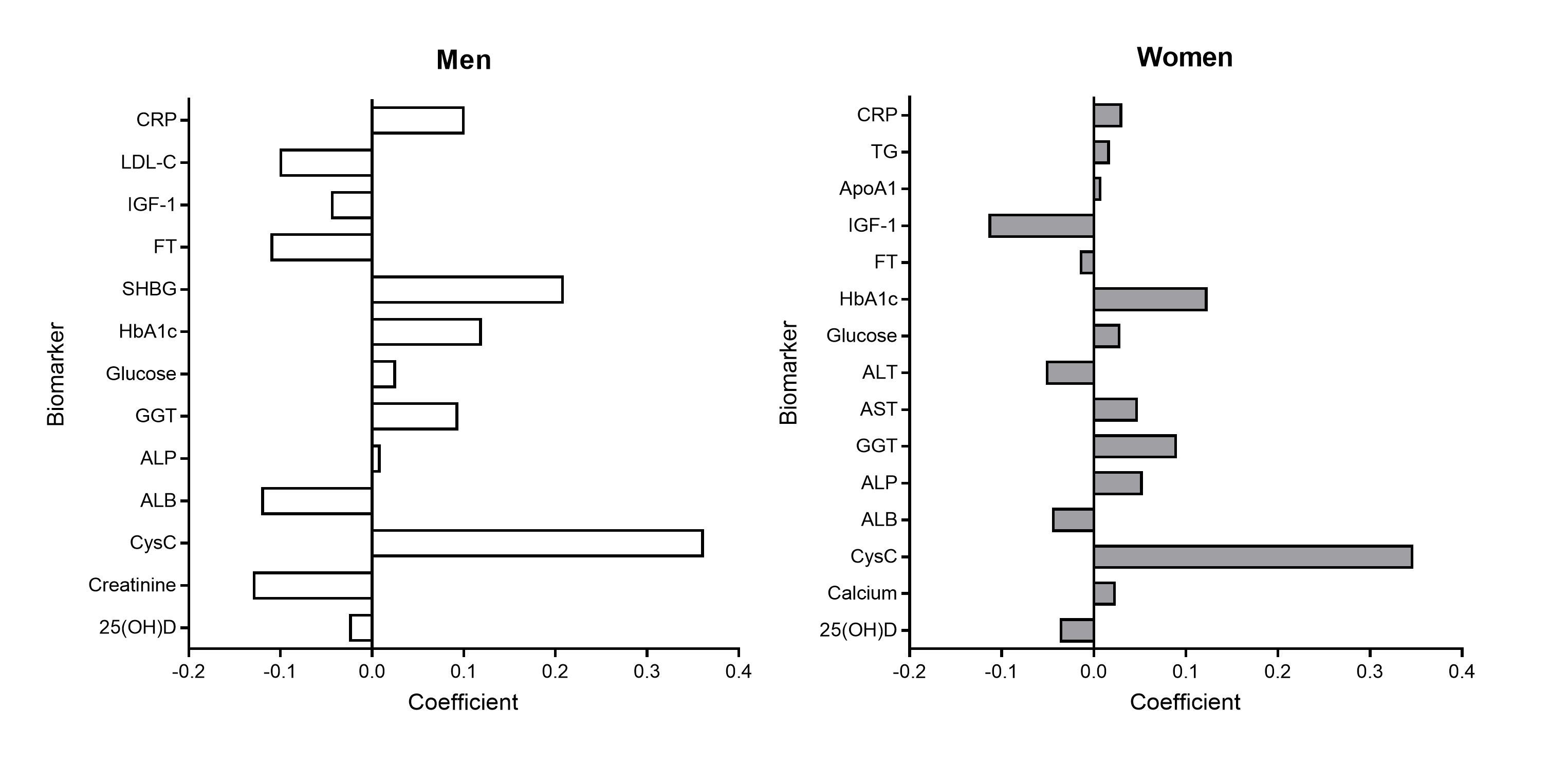

Supplement: Supplementary file 11 — Additional file 11: Figure S5. The coefficients of selected biomarkers derived from the LASSO regression for all-cause mortality in the training set after excluding 584 men and 259 women within two years of follow-up time. [file 12967_2023_4334_MOESM11_ESM.tif]

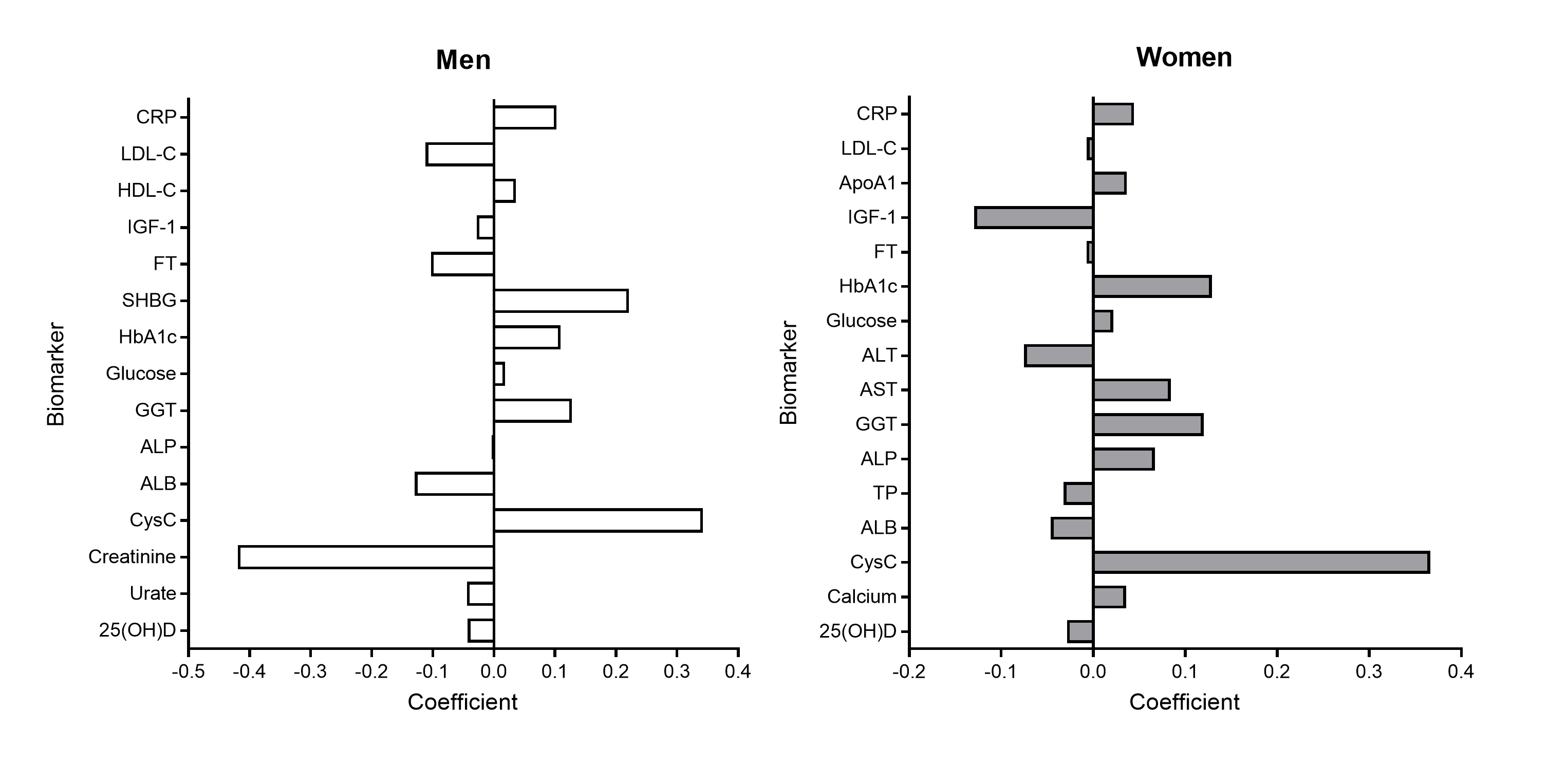

Supplement: Supplementary file 12 — Additional file 12: Figure S6. The coefficients of selected biomarkers derived from the LASSO regression for all-cause mortality in the training set after excluding 54,428 men and 42,998 women with estimated glomerular filtration rate < 90 mL/min/1.73 m2. [file 12967_2023_4334_MOESM12_ESM.tif]

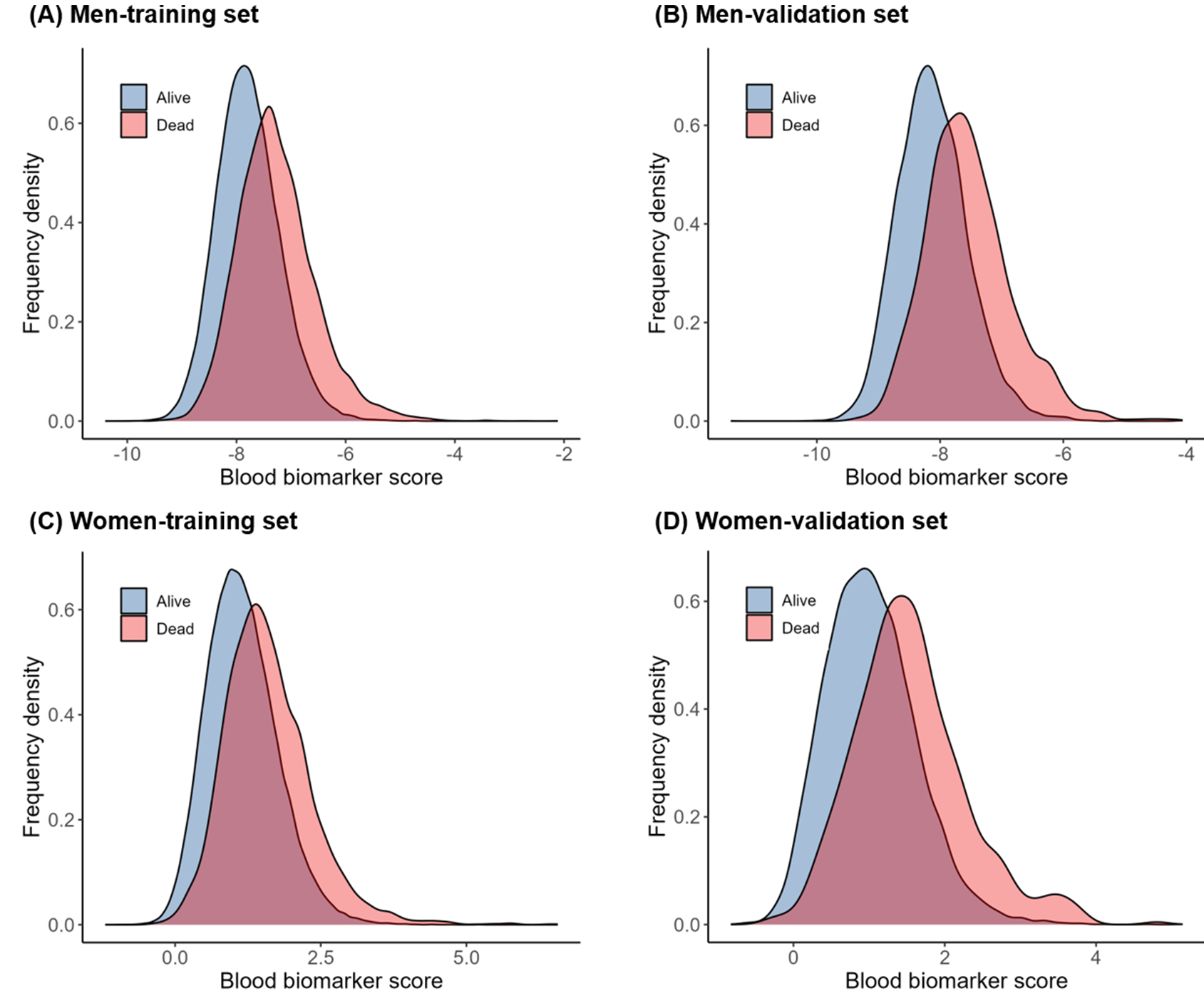

Supplement: Supplementary file 13 — Additional file 13: Figure S7. The distribution of the blood biomarker score between participants dead and alive in the training and validation sets. [file 12967_2023_4334_MOESM13_ESM.tif]

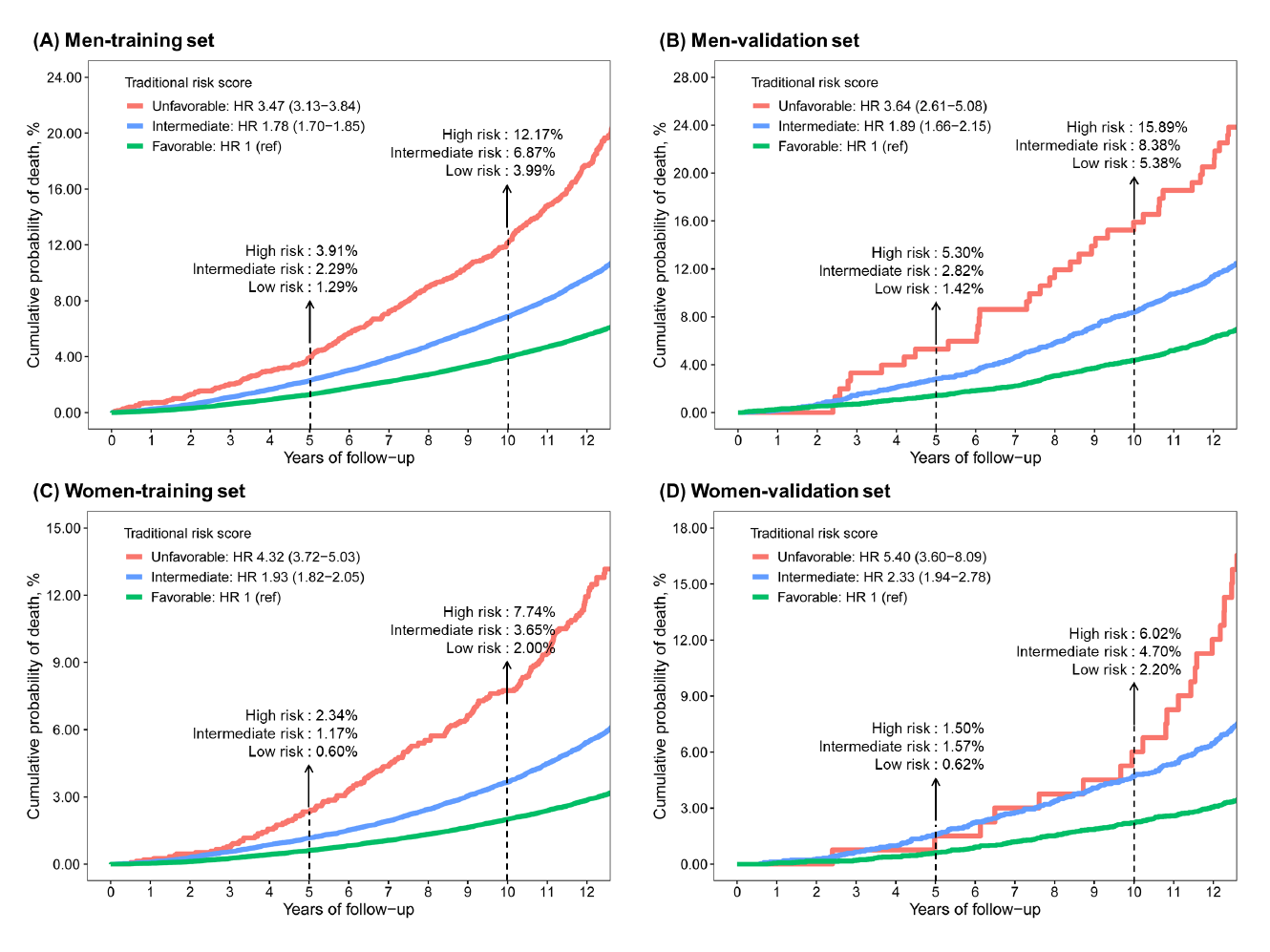

Supplement: Supplementary file 14 — Additional file 14: Figure S8. Cumulative probability of death by the three groups of traditional risk score in the training and validation sets for men and women. [file 12967_2023_4334_MOESM14_ESM.tif]

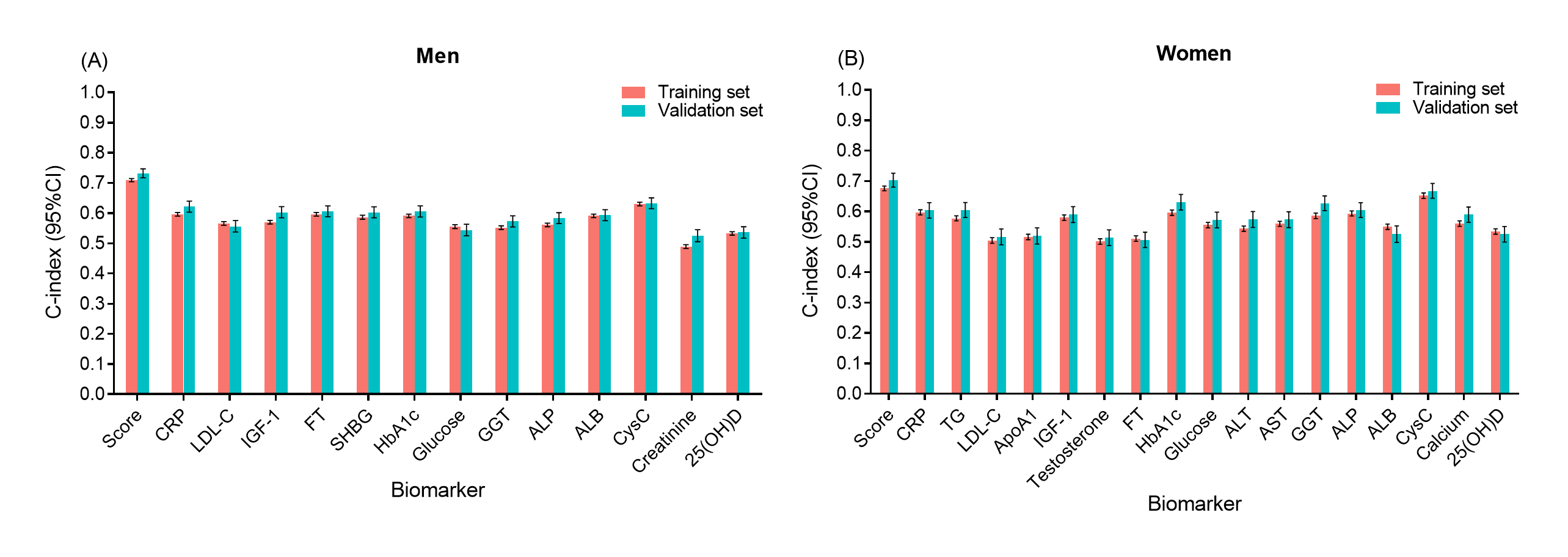

Supplement: Supplementary file 15 — Additional file 15: Figure S9. The C-index of predictors for all-cause mortality in the training and validation sets for men and women. [file 12967_2023_4334_MOESM15_ESM.tif]

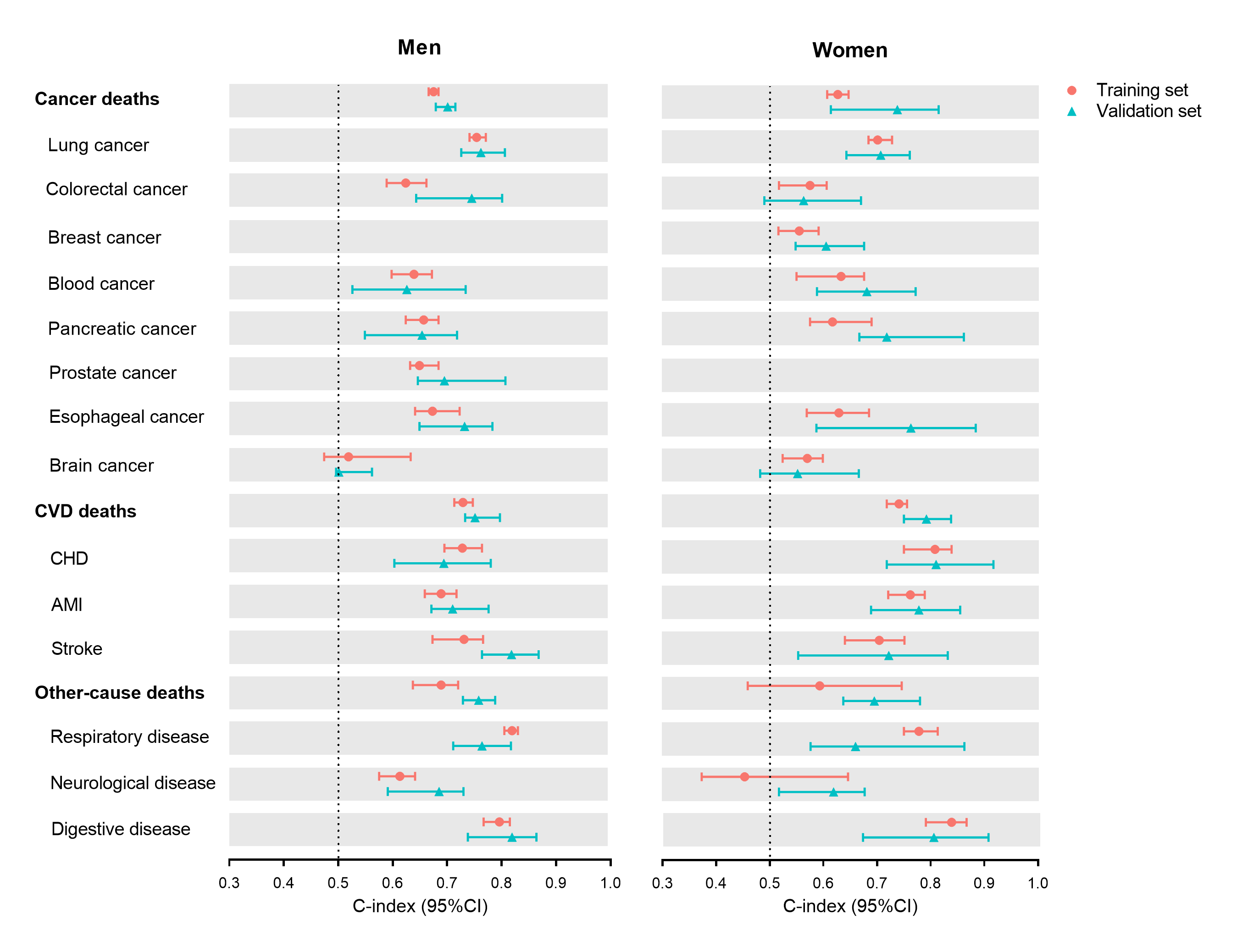

Supplement: Supplementary file 16 — Additional file 16: Figure S10. The C-index of blood biomarker scores for cause-specific mortality using competing risk models in the training and validation sets for men and women. [file 12967_2023_4334_MOESM16_ESM.tif]
